# Membrane-associated periodic skeleton regulates major forms of endocytosis in neurons through a signaling-driven positive feedback loop

**DOI:** 10.1126/sciadv.aeb0803

**Published:** 2026-02-11

**Authors:** Jinyu Fei, Yuanmin Zheng, Caden LaLonde, Yuan Tao, Ruobo Zhou

**Affiliations:** ^1^ Department of Chemistry, The Pennsylvania State University, University Park, PA 16802, USA.; ^2^ Department of Biomedical Engineering, The Pennsylvania State University, University Park, PA 16802, USA.; ^3^ Department of Biochemistry and Molecular Biology, The Pennsylvania State University, University Park, PA 16802, USA.; ^4^ Huck Institutes of Life Sciences, The Pennsylvania State University, University Park, PA 16802, USA.

## Abstract

Endocytosis enables neurons to internalize molecules, maintaining homeostasis and responsiveness. The neuronal membrane–associated periodic skeleton (MPS), an actin spectrin–based cytoskeletal lattice, is known to restrict clathrin-mediated endocytosis (CME) in axons, but its broader role in other neuronal compartments and endocytic pathways remains unclear. Here, we show that all four major endocytic pathways—CME, caveolin-, flotillin-, and fast endophilin–mediated endocytosis—are spatially gated by the MPS and occur exclusively within MPS-free “clearing” zones throughout all neuronal compartments. Disrupting the MPS broadly enhances both basal and ligand-induced endocytosis. We also identify a previously unknown feedback loop in which ligand-triggered endocytosis activates extracellular signal–regulated kinase signaling, promoting protease-mediated spectrin cleavage and MPS disruption, which in turn facilitates further endocytosis. Furthermore, the MPS limits amyloid precursor protein endocytosis, thereby suppressing Aβ42 production and linking MPS integrity to neurodegeneration. Our findings establish the MPS as a dynamic, signal-responsive modulator coupling membrane trafficking with cortical cytoskeletal organization and neuronal health.

## Teaser

A signal-responsive membrane skeleton gates endocytosis and ties membrane remodeling to neuronal health and Alzheimer’s disease.

## INTRODUCTION

Endocytosis is a fundamental and evolutionarily conserved cellular process, essential for a wide range of organisms, including yeast, animals, and plants (*1*). It plays a critical role in maintaining the homeostasis of cell membrane constituents by recycling specific lipid and protein components of the plasma membrane and in facilitating the cell’s nutrient uptake and communication with its extracellular environment and neighboring cells by internalizing and sampling extracellular materials, such as fluids, biomolecules, and microbes. In neurons, endocytosis regulates nerve cell migration and adhesion during brain development, establishes neuronal differentiation and axon-dendrite polarity, and governs synaptic activity and plasticity by controlling synaptic transmission and shaping the size, structure, and number of synapses, thereby influencing brain development, functions, and activities such as learning and memory (*2*). Conversely, dysregulation of endocytosis has been implicated in the pathogenesis of various neurological disorders (*3*–*5*). Genetic studies have identified mutations that alter the expression of endocytic machinery genes in neurodegenerative diseases such as Alzheimer’s disease (AD), Parkinson’s disease, and amyotrophic lateral sclerosis (*6*).

The regulation of endocytosis relies on the intricate interplay of protein-specific interactions, signaling pathway activation, and cytoskeleton rearrangement. While notable progress has been made in understanding the molecular mechanisms underlying endocytic pathways (*1*, *7*), many aspects of the molecular mechanisms underlying neuronal endocytosis remain poorly understood. To facilitate the uptake of diverse extracellular materials, various distinct endocytic mechanisms have been identified in nonneuronal cells, including clathrin-mediated endocytosis (CME, a clathrin- and dynamin-dependent pathway), lipid raft–mediated endocytosis (LRME, a clathrin-independent but dynamin-dependent pathway), fast endophilin–mediated endocytosis (FEME, a clathrin-independent but dynamin-dependent pathway for rapid ligand-driven internalization of specific membrane proteins), macropinocytosis, and phagocytosis (*1*, *7*). Among these, CME is the most well-studied endocytic pathway in neurons and has been shown to occur ubiquitously across nearly all neuronal subcompartments, including the soma, axon shaft, dendrite shaft, and both pre- and postsynaptic sites. In contrast, much less is known about the localization and regulation of other endocytic pathways in neurons. These pathways are functionally complementary rather than redundant: Although they can internalize overlapping membrane regions, each is defined by distinct molecular machineries, cargo preferences, regulatory mechanisms, temporal dynamics, and their precise contributions to neuronal homeostasis, plasticity, and signaling (*8*–*13*), underscoring their unique and essential roles within the complex neuronal environment.

A decade ago, super-resolution imaging enabled the discovery of the neuronal actin spectrin–based membrane-associated periodic skeleton (MPS), a structure in which actin filaments are organized into “rings” connected by spectrin tetramers, forming a one-dimensional (1D) periodic lattice with ~190-nm spacing beneath the plasma membrane of neurites (*14*). In mature neurons, the MPS extends throughout ~90% of axonal regions, including both the axon initial segment (AIS) and distal axons (*14*–*16*), and is also observed in ~50% of dendritic regions (*15*, *17*, *18*). The MPS is broadly distributed across diverse neuronal types, including excitatory and inhibitory neurons in both the central and peripheral nervous systems (*19*, *20*), and is conserved across species ranging from *Caenorhabditis elegans* to humans (*20*). This submembrane cytoskeletal network plays a critical role in organizing transmembrane proteins, such as ion channels, adhesion molecules, membrane transporters, and receptors (*14*, *15*, *17*, *21*–*24*). The MPS has been implicated in enhancing axonal stability under mechanical stress (*14*, *25*), mediating mechanosensation (*26*), controlling neurite diameter and bundling (*27*), influencing axon degeneration (*28*, *29*), and regulating neuronal receptor signaling (*23*) and CME (*23*, *30*), emphasizing its essential role in neuronal function and health.

A recent study has reported a previously unidentified cell surface–localized structure related to the MPS in neurons, termed “clearings,” which regulates CME at the AIS and proximal axon segments near the AIS (*30*). These clearings are empty, membrane-associated holes surrounded by the actin spectrin–based MPS network. Clathrin-coated pits (CCPs) preferentially form at the center of these preexisting clearings and are stabilized as long-lived and stalled structures before undergoing scission and conclusive endocytosis in response to plasticity-inducing stimulation. Despite these insights, several critical questions remain unresolved. First, although the initial study reports that the clearings of the MPS scaffold exclusively formed at AIS and proximal axon next to AIS and were absent from soma and dendrites, this observation was made using relatively immature neurons at 14 days in vitro (DIV 14), when the MPS has not fully developed in the somatodendritic compartments (*18*). Our previous systematic investigation of the developmental progression of the MPS demonstrated that MPS formation occurs much slower in somatodendrites than in axons, with the MPS network density in soma and dendrites reaching saturation only around DIVs 21 to 28 (*18*). Therefore, it remains an open question whether similar CCP-containing clearings exist in distal axons or somatodendritic compartments in more mature (DIV 21 or older) neurons. Second, whether clathrin-independent endocytic pits, such as those involved in LRME or FEME, also preferentially localize to the centers of MPS-free clearings, and whether their endocytic activity is similarly regulated by the MPS, have not been investigated. Third, the mechanisms that control the formation, size, and spatial distribution of these clearings in response to increased endocytic demand, such as during neuronal stimulation, are largely unexplored. Given that the MPS inhibits ligand-induced CME, a critical unresolved question is how the MPS is locally disassembled, through the formation or expansion of clearings, to accommodate elevated endocytic activity during periods of rapid neuronal signaling.

Here, using super-resolution fluorescence microscopy (*31*, *32*) in combination with quantitative cell biology approaches, we systematically investigated four major types of endocytosis in neurons: CME, caveolin-mediated endocytosis and flotillin-mediated endocytosis (*33*) (two prominent forms of LRME), and FEME (*12*). Similar to CME, we found that caveolin-mediated endocytosis, flotillin-mediated endocytosis, and endophilin-mediated endocytosis (i.e., FEME) can occur broadly across nearly all neuronal compartments, including the soma, AIS, axon shaft, and dendrite shaft. Notably, we identified CCP-centered clearings not only in the proximal axon shaft as previously reported (*30*) but also in distal axons and somatodendritic regions of mature neurons. For the other three types of endocytic pathways, we identified analogous clearings formed by the actin spectrin–based MPS, with caveolin-, flotillin-, or endophilin-enriched nanodomains located at their centers, resembling the previously reported CCP-centered clearings. Disruption of the neuronal MPS enhanced both basal endocytosis mediated by clathrin, caveolin, flotillin, and endophilin, as well as ligand-induced receptor endocytosis across these pathways. These findings suggest that the MPS serves as a physical barrier to suppress endocytosis for all the four endocytosis types. We further demonstrated that ligand-induced receptor endocytosis mediated by these proteins activates downstream extracellular signal–regulated kinase (ERK) signaling cascades and proteases (i.e., calpains and caspases), which in turn can promote MPS disassembly (i.e., enlarge the total area of clearings), forming a positive feedback loop that further enhances endocytosis. These findings indicate that the MPS acts as a dynamically regulated barrier to modulate endocytosis rates. Last, we demonstrated that the MPS exerts a neuroprotective role by negatively regulating amyloid precursor protein (APP) endocytosis through the same positive feedback loop. Given that APP endocytosis is up-regulated during neuronal aging and in AD (*3*, *4*), this MPS-mediated regulation may serve as a protective mechanism against disease-associated endocytic dysregulation. Together, these findings provide structural and mechanistic insights into the regulation of diverse neuronal endocytosis pathways under both physiological and pathological conditions.

## RESULTS

### Compartment-resolved mapping of four endocytic pit types reveals broad suppression of basal endocytosis by the neuronal MPS

As studies of neuronal endocytosis have primarily focused on CME and it remains largely unclear which neuronal compartments support other endocytic pathways, we first sought to develop immunofluorescence (IF)–based assays capable of accurately mapping the spatial distributions of four major types of endocytic pits, including the endocytic pits for CME (i.e., CCPs), caveolin-mediated endocytosis, flotillin-mediated endocytosis, and FEME, respectively, across different neuronal compartments. In cells, CME serves as the major constitutive “housekeeping” pathway, continuously sampling the extracellular environment and mediating the bulk uptake of diverse cargoes such as nutrients, antigens, and signaling receptors. It is critical for maintaining cellular homeostasis and regulating signal transduction across various cell types (*8*). Caveolin-mediated endocytosis is less involved in bulk uptake and more specialized in organizing signal transduction and lipid regulation through flask-shaped membrane invaginations called caveolae. It specializes in internalizing specific lipids and certain toxins (*9*, *10*). Flotillin-mediated endocytosis functions through clathrin- and caveolin-independent, microdomain-associated vesicles formed by flotillin scaffolds. This pathway mediates the uptake of selected membrane proteins and contributes to cytoskeletal organization and receptor recycling (*11*). FEME represents a rapid, activity-dependent route that enables the efficient internalization of activated surface receptors upon ligand binding, thereby supporting swift cellular responses and dynamic signaling modulation (*12*, *13*). Together, these pathways form a complex yet complementary network that allows cells to precisely regulate the internalization of external materials and membrane proteins, facilitating adaptation to diverse physiological demands and environmental cues. Consequently, mapping their spatial distribution across different neuronal subcompartments can provide a comprehensive framework for understanding the endocytic landscape in neurons. While the IF staining protocol for visualizing CCPs in neurons has been well documented (*30*), staining protocols for other endocytic pits in neurons are underdeveloped. Previous studies have reported that the commonly used cell permeabilization method using Triton X-100 substantially alters the staining patterns of lipid-raft–mediated endocytic pits (*34*). To overcome this, we expressed moderate level of green fluorescent protein (GFP)–tagged caveolin-1 (Cav1), flotillin-1 (Flot1), or endophilin-A2 (EndoA2) in cultured neurons through low-titer lentiviral transduction, and tested IF protocols previously developed for nonneuronal systems (fig. S1) (*12*, *33*–*35*). Neurons were immunostained with IF-validated antibodies against endogenous Cav1 (*35*), Flot1 (*33*), and EndoA2 (*12*), respectively. The observed strong colocalization between immunostained puncta and the puncta in the GFP channel, which are likely endocytic pits, confirmed the specificity of these antibodies and validated the IF-based assays for visualizing these endocytic pits in neuronal systems.

**Fig. 1.**
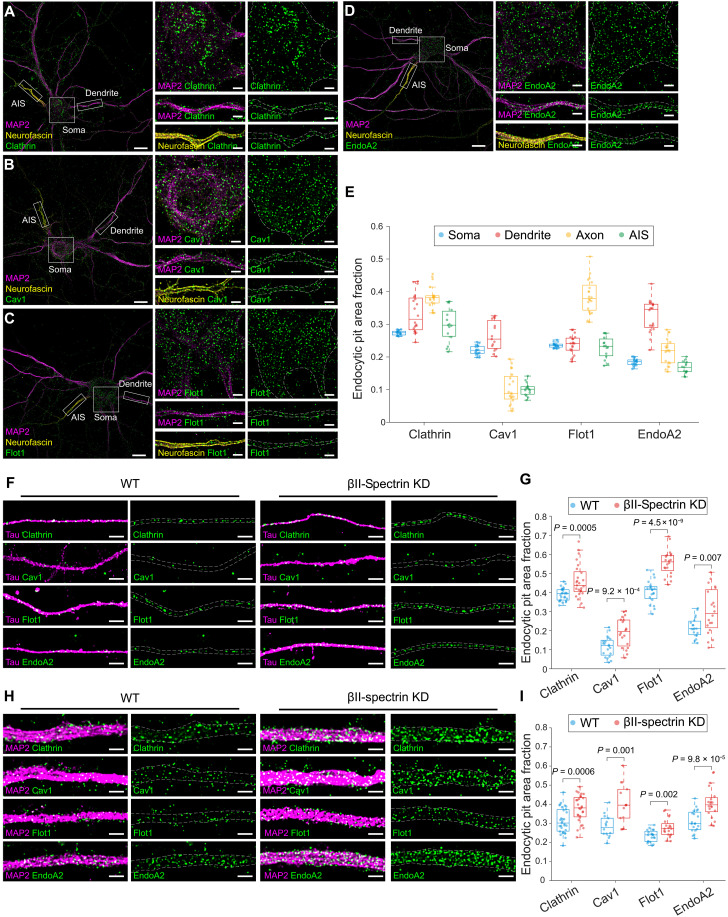
Compartment-resolved mapping of four endocytic pit types reveals broad suppression of basal endocytosis by the MPS in neurons. (**A** to **D**) Left: Stitched SIM images showing the distributions of endogenous endocytic pits, clathrin (A), Cav1 (B), Flot1 (C), or EndoA2 (D) in WT neurons. Endocytic pits are shown in green, with compartment markers MAP2 (magenta) and neurofacsin (yellow). Scale bar, 10 μm. Right: Enlarged SIM images of the three boxed regions on the left, corresponding to soma, dendrite, and AIS compartments, respectively. Scale bar, 2 μm. (**E**) Boxplots showing the area fraction of endogenous endocytic pits in different compartments of WT neurons. (**F**) Left: SIM images of tau (magenta) and endogenous endocytic pits (green) in distal axons of WT neurons. Right: The same as on the left, but in βII-spectrin KD neurons. Scale bars, 2 μm. (**G**) Boxplots showing the area fraction of endogenous endocytic pits (green) in distal axons of WT and βII-spectrin KD neurons. (**H**) Left: SIM images of MAP2 (magenta) and endogenous endocytic pits (green) in dendrites of WT neurons. Right: The same as on the left, but in βII-spectrin KD neurons. Scale bars, 2 μm. (**I**) Boxplots showing the area fraction of endogenous endocytic pits in dendrites of WT and βII-spectrin KD neurons.

Using these IF-based assays, with immunostaining for endogenous clathrin, Cav1, Flot1, and EndoA2, and structured illumination microscopy (SIM) (*36*, *37*), a super-resolution imaging method with a lateral resolution of 100 to 120 nm, we investigated the basal-level spatial distributions of the four major types of endocytic pits across distinct neuronal compartments, including the AIS, distal axons, soma, and dendrites. To accurately distinguish these neuronal compartments, we used neurofascin, tau, and MAP2 as markers for the AIS, distal axonal segments, and dendrites, respectively (Fig. 1, A to D, and fig. S2, C to F). We quantified basal-level densities of these endocytic pits using endocytic pit area fraction, defined as the ratio of total endocytic pit area to the corresponding compartmental area demarcated by these markers. We found that all four types of endocytic pits were present in all neuronal compartments examined. CCPs and Flot1-pits were denser in distal axons than in any other neuronal compartments, whereas Cav1- and EndoA2-pits were denser in dendrites compared to other neuronal compartments (Fig. 1E), indicating that these four endocytic pathways are differentially regulated across distinct neuronal compartments.

Having established the basal-level distributions of these endocytic pits across different neuronal compartments, we next aimed to examine the regulatory role of the MPS in modulating these distributions. To study how MPS disruption affects the basal-level distributions and densities of the four types of endocytic pits, we transduced neurons with adenovirus expressing short hairpin RNA (shRNA) against βII-spectrin, a key structural component of the MPS, to disrupt the MPS both in the axonal and somatodendritic compartments, as described previously (*15*, *18*, *23*, *27*). The βII-spectrin expression level in βII-spectrin knockdown (KD) neurons decreased by ~70%, compared to wild-type (WT) neurons, which were transduced with adenovirus expressing scrambled control shRNA as a control (fig. S2, A and B). The immunostained patterns for endogenous CCPs, Cav1-pits, Flot1-pits, and EndoA2-pits in mature (DIVs 21 to 28) WT and βII-spectrin KD neurons showed that MPS disruption significantly increased the densities of all four types of endocytic pits in all neuronal compartments examined including AIS, distal axonal segments, and dendrites, compared to WT neurons (fig. S2, G and H, and Fig. 1, F to I). To determine whether such enhancement of endocytosis observed in mature βII-spectrin KD neurons reflected MPS disruption rather than MPS-independent effects of βII-spectrin depletion, we analyzed younger neurons (DIV 7), a developmental stage at which the dendritic MPS is not yet established (*18*). In these immature neurons, endocytic pit densities were comparable between WT and βII-spectrin KD neurons, suggesting that the enhanced pit densities observed in mature βII-spectrin KD neurons are likely due to MPS disruption rather than other MPS-independent effects (fig. S2, I and J). Together, these results suggest that the MPS functions as a physical barrier to suppress basal endocytosis across all four endocytic pathways in all neuronal compartments, likely by acting as a physical barrier that restricts endocytic pit formation.

### Four major types of endocytic pits are localized within clearings of the MPS lattice in axons

**Fig. 2.**
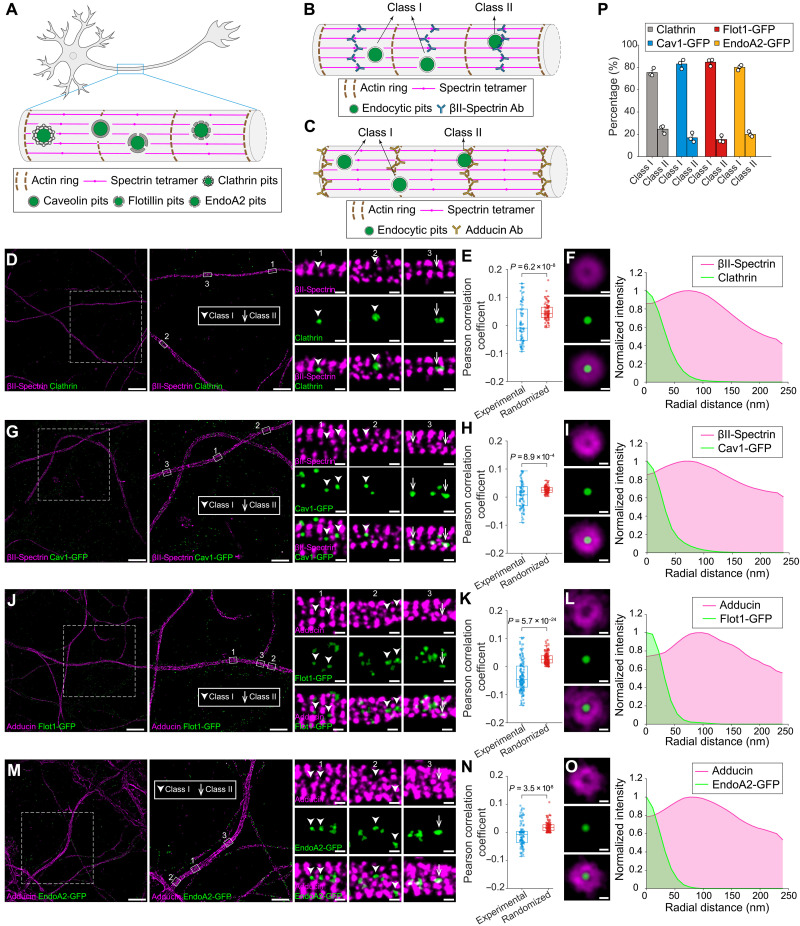
Four major types of endocytic pits are localized within clearings of the MPS lattice in axons. (**A**) Schematic illustrating the spatial distributions of clathrin, Cav1, Flot1, and EndoA2 endocytic pits, relative to periodic MPS lattice in axons. (**B**) Schematic illustrating two distinct types of endocytic pits based on their spatial positioning relative to periodic βII-spectrin lattice in axons. Class I pits do not overlap with MPS lattice, whereas class II do. The MPS was visualized by immunostaining with antibodies targeting the C terminus of βII-spectrin, which mark the centers of spectrin tetramers. (**C**) The same as in (B) but showing spatial relationships with the periodic adducin lattice in axons. The MPS was visualized by immunostaining with antibodies targeting the adducin, which mark the terminal ends of spectrin tetramers. (**D**) Left: Dual-color STORM images of βII-spectrin (magenta) and endogenous clathrin (green) in axons. Right: Magnified views of class I and class II CCPs in the boxed regions. Scale bars, 10 μm (left), 5 μm (middle), and 200 nm (right). (**E**) PCCs between βII-spectrin and endogenous clathrin under experimental and randomized conditions. (**F**) Left: Averaged dual-color STORM images of βII-spectrin (magenta) and endogenous clathrin (green), generated by aligning individual STORM images to the centers of CCPs. Right: Radial intensity profiles of the averaged images shown on the left. Scale bar, 100 nm. (**G** to **I**) The same as in (D) to (F) but for βII-spectrin (magenta) and exogenously expressed Cav1 (green). (**J** to **L**) The same as in (D) to (F) but for adducin (magenta) and exogenously expressed Flot1 (green). (**M** to **O**) The same as in (D) to (F) but for adducin (magenta) and exogenously expressed EndoA2 (green). (**P**) Percentages of class I and class II pits for endogenous clathrin, exogenously expressed Cav1, exogenously expressed Flot1 and exogenously expressed EndoA2 in axons.

We further investigated the structural basis underlying the MPS’s inhibitory role in regulating four major types of endocytosis in axons. To resolve the spatial relationship between endocytic pits and the MPS, we used dual-color 3D stochastic optical reconstruction microscopy (STORM) (*36*, *38*), a super-resolution imaging technique with lateral resolution of 20 to 30 nm and axial resolution of 50 to 60 nm (Fig. 2A). The MPS was visualized in cultured mature neurons (at or after DIV 21) through immunostaining of the C terminus of βII-spectrin or adducin, which mark the periodic rings formed by spectrin tetramer centers or actin filaments, respectively. Endocytic pits were immunostained using either antibodies against endogenous endocytic proteins, or an anti-GFP antibody for labeling the exogenously expressed GFP-tagged endocytic proteins through low-titer lentiviral transduction.

3D STORM cross sections (*y*/*z* views) revealed that all four types of endocytic pits were present both at the plasma membrane and within axonal shafts (fig. S3A), with the fractions of endogenous endocytic pits being localized at the cell surface being 78.9 ± 4.0, 82.3 ± 1.8, 67.2 ± 1.7, and 67.0 ± 6.9 for CCPs, Cav1-pits, Flot1-pits, and EndoA2-pits, respectively (fig. S3B). The surface-localized fractions of GFP-tagged endocytic pits closely resembled those of their endogenous counterparts, except for GFP-tagged Cav1 pits, which displayed a slightly lower surface-localized fraction, suggesting that both labeling strategies yield comparable results. To further validate our quantification method, we treated neurons with dyngo-4a, a potent dynamin inhibitor that prevents CCP scission from the plasma membrane (*39*), which led to an expected increase in the fraction of surface-localized CCPs in both axonal and dendritic compartments, confirming the reliability of our measurements (fig. S3D).

Next, we quantified the diameters of all four types of endogenous endocytic pits localized at the plasma membrane, which averaged 82.5 ± 2.8, 75.0 ± 4.3, 71.7 ± 2.8, and 67.0 ± 3.4 nm for CCPs, Cav1-pits, Flot1-pits, and EndoA2-pits, respectively (fig. S3C). The average diameters for the internalized fractions of the four endogenous endocytic pits were similar to those of surface-localized pits, indicating that most surface-localized puncta analyzed are likely fully assembled endocytic pits (fig. S3, C and E). In addition, GFP-tagged endocytic pits exhibited similar diameters to their endogenous counterparts, for both surface-localized and internalized fractions (fig. S3C).

For the surface-localized fractions of these four types of endocytic pits, we examined their spatial distributions relative to the MPS in axons by classifying the relative positions of endocytic pits into two scenarios (Fig. 2, B and C): (Class I) The pit has no overlap with the MPS, and (class II) the pit overlaps with the MPS. Quantification revealed that class I pits were much more prevalent than class II pits for all four types of endocytic pits, suggesting that all four types of surface-localized pits are spatially excluded from the MPS (Fig. 2, D, G, J, M, and P). To confirm this exclusion, we calculated the Pearson’s correlation coefficients (PCCs) between the surface-localized pits and the MPS across 70 to 130 axonal regions and found that these experimental coefficient values were significantly lower than those calculated from randomization controls, where the positions of all pits in each analyzed axonal region were randomly shuffled before calculating the PCC (Fig. 2, E, H, K, and N). As negative controls, we labeled neurons with two widely used membrane markers wheat germ agglutinin (WGA) and cholera toxin subunit B (CTB), and imaged their spatial distributions relative to the MPS in axons using STORM, following the same procedure as for the endocytic pits. The calculated PCCs between membrane marker and the MPS were not significantly different from those calculated from randomized controls, supporting the observed spatial exclusion between pits and the MPS as a true biological pattern (fig. S4, A to D). In addition, we compared the quantification results obtained from endogenously labeled pit proteins with those obtained from exogenously expressed GFP-tagged pit proteins (fig. S5, A to G). Both labeling strategies yielded comparable results. Last, to better visualize the average spatial pattern of these exclusion zones, we generated averaged dual-color STORM images by aligning individual cropped images centered on each endocytic pits, without reorienting neurites to the same MPS periodic axis. For all four types of pits, the corresponding radial fluorescence intensity profiles revealed that these pits are located in regions devoid of MPS immunostaining (Fig. 2, F, I, L, and O). These circular clearing structures observed closely resembled those CCP-centered clearings previously observed at the AIS (*30*). These MPS-surrounding circular clearings in the averaged dual-color STORM images were consistently observed regardless of the MPS labeling strategy (fig. S4, E to K), whether visualized via the C terminus of βII-spectrin (marking spectrin tetramer centers) or adducin (marking spectrin tetramer ends), indicating that endocytic pits are excluded from the entire MPS-occupied membrane region. Together, these findings demonstrate that CCP-centered clearings are not only present at or near the AIS but also extend into distal axonal segments, and similar clearings are also present in the axons for the three clathrin-independent endocytic pits examined.

### Four major types of endocytic pits are localized within clearings of the MPS lattice in dendrites

The initial clearing study reported that CCP-centered clearings observed at the AIS were absent from somatodendritic compartments (*30*). However, this absence may be attributed to imaging being performed on DIV-14 neurons, while the MPS develops much more slowly in somatodendrites than in axons (*18*). The MPS coverage across the plasma membrane of somatodendrites reaches only ~50% saturation around DIV 21 to 28, whereas MPS coverage in axons reaches ~90% by DIV 14. This developmental delay raises the possibility that similar clearings may emerge in somatodendrites of more mature neurons (DIV 21 or older).

**Fig. 3.**
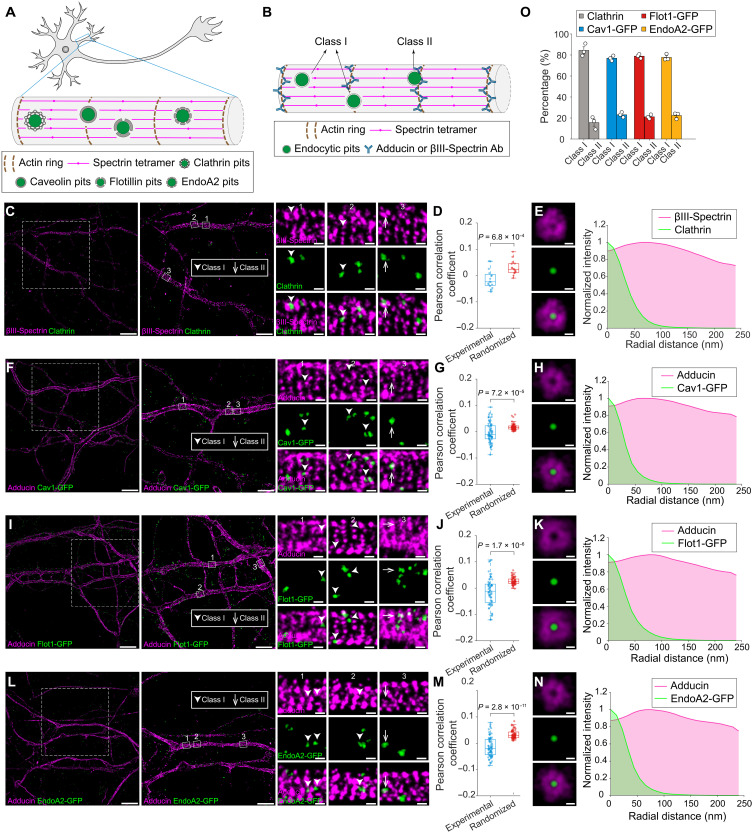
Four major types of endocytic pits are localized within clearings of the MPS lattice in dendrites. (**A**) Schematic illustrating the spatial distribution of clathrin, Cav1, Flot1, and EndoA2 endocytic pits, relative to periodic MPS lattice in dendrites. (**B**) Schematic illustrating two distinct types of endocytic pits based on their spatial positioning relative to periodic βIII-spectrin or adducin lattice in dendrites. Class I pits do not overlap with MPS lattice, whereas class II pits do. The MPS was visualized by immunostaining with antibodies targeting the N terminus of βIII-spectrin or adducin, which both mark terminal ends of spectrin tetramers. (**C**) Left: Dual-color STORM images of βIII-spectrin (magenta) and endogenous clathrin (green) in dendrites. Right: Magnified views of class I and class II CCPs in the boxed regions. Scale bars, 10 μm (left), 5 μm (middle), 200 nm (right). (**D**) PCCs between βIII-spectrin and endogenous clathrin under experimental and randomized conditions. (**E**) Left: Averaged dual-color STROM images of βIII-spectrin (magenta) and endogenous clathrin (green), generated by aligning individual STORM images to the centers of CCPs. Right: Radial intensity profile of averaged images shown on the left. Scale bar, 100 nm. (**F** to **H**) The same as in (C) to (E) but for adducin (magenta) and exogenously expressed Cav1 (green). (**I** to **K**) The same as in (C) to (E) but for adducin (magenta) and exogenously expressed Flot1 (green). (**L** to **N**) The same as in (C) to (E) but for adducin (magenta) and exogenously expressed EndoA2 (green). (**O**) Percentages of class I and class II endocytic pits for endogenous clathrin, exogenously expressed Cav1, exogenously expressed Flot1, and exogenously expressed EndoA2 in dendrites.

Since the MPS coverage is comparable between the soma and dendrites in mature neurons, and STORM imaging is technically easier in dendrites, we selected dendrites as the representative somatodendritic compartment for our imaging analysis (Fig. 3A). We first examined the spatial relationship between each of the four endocytic pit types and the surface-localized MPS in the radial direction within cross-sectional images of dendrites. The dendritic MPS was labeled by immunostaining either adducin or the N terminus of βIII-spectrin (a β-spectrin isoform present primarily in somatodendrites but not axons), both of which mark the periodic rings formed by actin filaments (Fig. 3B). Endocytic pits were immunostained using the same two labeling strategies used for axon imaging. 3D STORM cross sections (*y*/*z* views) revealed that all four types of endocytic pits were present both at the plasma membrane and within dendritic shafts (fig. S3F), with the surface-localized fractions being similar to those observed in axons (fig. S3G). The average diameters of the internalized endocytic pits closely matched those of the surface-localized fractions, and these average diameters determined in dendrites were comparable to those observed in axons. Both labeling strategies for endocytic pits produced consistent results (fig. S3H).

We then examined the spatial relationship between each type of endocytic pit (restricted to the surface-localized fractions of the four pit types) and the MPS in top-view STORM images of dendrites. As in the axon analysis, surface-localized endocytic pits were classified into two classes based on their spatial overlap with the MPS. Two-color STORM revealed that class I pits (i.e., pits without overlap with the MPS) were markedly more prevalent than class II pits for all four types of endocytic pits (Fig. 3, C, F, I, L, and O, and fig. S5, H, J, L, and N). The spatial exclusion pattern of surface-localized endocytic pits from the MPS was confirmed by Pearson’s correlation analysis as performed for axons, where the experimental coefficients calculated from experimental images were significantly lower than those calculated from the corresponding randomization controls (Fig. 3, D, G, J, and M, and fig. S5 I, K, and M). Last, intensity profiles from averaged STORM images aligned to the centers of endocytic pits revealed circular clearings surrounding clathrin-, Cav1-, Flot1- or EndoA2-endocytic pits in dendritic compartments (Fig. 3, E, H, K, and N). Together, these results suggest that circular clearings are a conserved structure in both axonal and somatodendritic compartments of mature neurons, with both clathrin-dependent and clathrin-independent endocytic pits residing at their centers.

### MPS inhibits ligand-induced endocytosis in both axonal and somatodendritic compartments

**Fig. 4.**
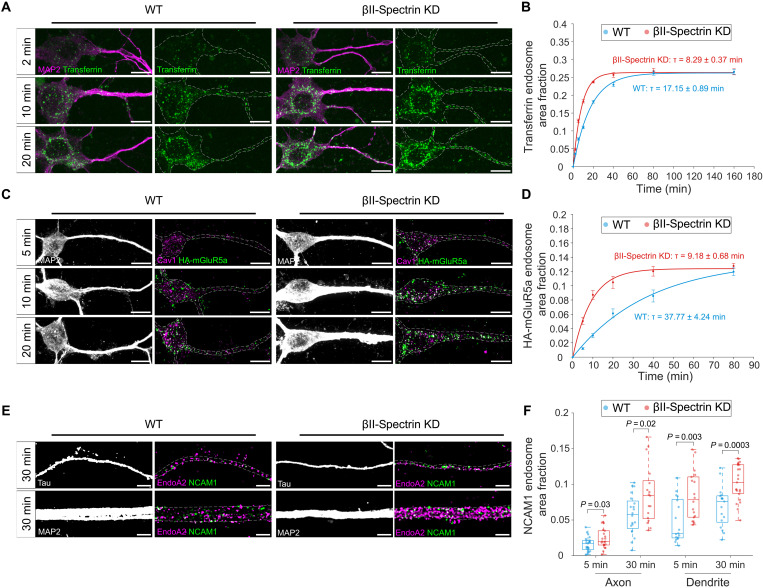
MPS inhibits ligand-induced endocytosis in both axonal and somatodendritic compartments. (**A**) Confocal fluorescence images of MAP2 (magenta) and internalized CF568-transferrin (green) in somatodendritic region of WT or βII-spectrin KD neurons treated with CF568-transferrin for 2, 10, and 20 min. Scale bars, 10 μm. (**B**) Time course of CF568-transferrin endocytosis in somatodendritic regions of WT and βII-spectrin KD neurons, quantified by the area fraction of transferrin-positive endosomes. Solid lines represent single-exponential fits to the data. (**C**) Confocal fluorescence images of MAP2 (gray), internalized HA-mGluR5a (green), and endogenous Cav1 (magenta) in somatodendritic region of WT or βII-spectrin KD neurons overexpressing HA-mGluR5a and treated with anti-HA antibody for 5, 10, and 20 min. Scale bars, 10 μm. (**D**) Time course of Cav1-mediated HA-mGluR5a endocytosis in the somatodendritic regions of WT and βII-spectrin KD neurons, quantified by the area fraction of Cav1-positive HA-mGluR5a endosomes. Solid lines represent single-exponential fits to the data. (**E**) SIM images of internalized NCAM1 (green) and endogenous EndoA2 (magenta) in axonal (top) and somatodendritic (bottom) regions of WT or βII-spectrin KD neurons treated with anti-NCAM1 antibody for 30 min. Scale bars, 2 μm. (**F**) Boxplots of EndoA2-mediated NCAM1 endocytosis in axonal and somatodendritic regions of WT and βII-spectrin KD neurons, quantified by the area proportion of EndoA2-positive NCAM1 endosomes.

Given our observation that MPS disruption significantly increased the basal endocytosis of the four major types across all neuronal compartments, we investigated whether the MPS similarly inhibits ligand-induced endocytosis mediated by clathrin, caveolin, flotillin, or endophilin—which are generally considered to occur more rapidly than basal-level, constitutive endocytosis. We first examined transferrin and low-density lipoprotein (LDL) endocytosis in neurons, both of which are forms of ligand-induced endocytosis primarily mediated by clathrin (i.e., CME) in most cell types (*40*, *41*). Incubating fluorophore-conjugated transferrin or LDL with cultured neurons resulted in an increased number of internalized transferrin or LDL over time in the somatodendrites but not axons, consistent with previous studies (*40*, *41*). To ensure that only internalized ligands were quantified, we performed an acid wash to remove surface-bound transferrin or LDL before imaging. To further validate the endocytic pathways involved in transferrin or LDL uptake, we pretreated neurons with four inhibitors targeting distinct pathways (*1*) and assessed their effects, including: (i) shRNA against clathrin heavy chain (CHC) to disrupt CME (*42*) with CHC KD confirmed by more than a 60% reduction of CHC IF intensity in CHC KD neurons compared to WT neurons (fig. S6, A and B); (ii) dyngo-4a, which inhibits CME, FEME, and certain types of LRME; (iii) nystatin, which disrupts lipid rafts and hence LRME by sequestering cholesterol; and (iv) 5-[*N*-ethyl-*N*-isopropyl] amiloride (EIPA), a Na^+^/H^+^ exchanger inhibitor that disrupts macropinocytosis but not receptor-mediated endocytosis. Transferrin uptake by neurons was inhibited by CHC KD, dyngo-4a, and EIPA but not by nystatin (fig. S6, C and D), suggesting that transferrin uptake depends on CME and possibly macropinocytosis. Consistent with this, imaging revealed that internalized transferrin strongly colocalized with clathrin but not with Cav1, Flot1, or EndoA2 (fig. S6, E and F), further supporting CME as the primary pathway of transferrin uptake. In contrast, LDL uptake was inhibited only by CHC KD and dyngo-4a, indicating that LDL uptake relies exclusively on CME (fig. S6, I and J). To determine whether MPS disruption affects clathrin-mediated transferrin and LDL uptake, we quantified the area fraction of internalized transferrin or LDL puncta, defined as the ratio of total transferrin or LDL puncta area to the corresponding dendritic area, in both WT and βII-spectrin KD neurons (Fig. 4A and fig. S6K). By fitting the puncta area fraction over time to a single-exponential curve, we determined the time constants for uptake. Such time-course analysis revealed that transferrin uptake was faster in βII-spectrin KD neurons with a time constant τ = 8.29 ± 0.37 min, compared to τ = 17.15 ± 0.89 min in WT neurons (Fig. 4B). Similarly, LDL uptake was enhanced in βII-spectrin KD neurons (τ = 35.25 ± 1.49 min), compared to WT neurons (τ = 54.63 ± 2.45 min) (fig. S6L). Notably, βII-spectrin KD did not alter surface expression levels of transferrin receptors (TfRs) or LDL receptors, ruling out increased surface expression levels of TfRs or LDL receptors as a cause for the accelerated transferrin or LDL-induced endocytosis (fig. S6, G, H, M, and N). Together, these findings indicate that the MPS acts as a physical barrier to restrict ligand-induced CME.

We next examined whether MPS disruption affects ligand-induced LRME. A previous study reported that in cultured neurons overexpressing hemagglutinin (HA)–tagged metabotropic glutamate receptor 5a (mGluR5a) with the HA epitope tag located on its extracellular N terminus (HA-mGluR5a), treatment with an anti-HA antibody induced HA-mGluR5a endocytosis across all neuronal compartments via a proposed lipid raft–mediated and clathrin-independent pathway (*43*). Using the same assay, treatment with the anti-HA antibody followed by an acid wash to remove surface-bound antibody resulted in a punctate immunostaining pattern of HA-mGluR5a, indicating its internalization. A subset of these puncta was colocalized with Cav1 pits but not with clathrin-, Flot1-, or EndoA2- pits (fig. S7, A and B), supporting a caveolin-mediated mechanism. To confirm the endocytic pathway involved, we tested the effects of the four inhibitors described above. Antibody-induced HA-mGluR5a endocytosis was significantly inhibited by dyngo-4a or nystatin, but not by CHC KD or EIPA, further indicating that HA-mGluR5a endocytosis occurs, at least partially, through caveolin-mediated endocytosis, a major LRME pathway (fig. S7, C and D). Next, we assessed the effect of MPS disruption on antibody-induced HA-mGluR5a endocytosis. WT and βII-spectrin KD neurons were incubated with the anti-HA antibody over a time course, followed by immunostaining for internalized HA-mGluR5a and endogenous Cav1. This allowed us to selectively analyze the fraction of internalized HA-mGluR5a that colocalized with endogenous Cav1-pitsa (Fig. 4C). Time-course analysis revealed that caveolin-mediated HA-mGluR5a endocytosis occurred faster in βII-spectrin KD neurons (τ = 9.18 ± 0.68 min) compared to WT neurons (τ = 37.77 ± 4.24 min) (Fig. 4D). This enhancement was not due to changes in the surface expression of HA-mGluR5a (fig. S7, E and F). Together, our data indicate that MPS disruption facilitates ligand-induced LRME.

Last, we assessed the impact of MPS disruption on ligand-induced FEME. When examining endogenous neural cell adhesion molecule 1 (NCAM1) internalization in mature neurons, triggered by treatment with a monoclonal anti-NCAM1 antibody against its extracellular domain, we found that the internalized NCAM1 puncta colocalized with EndoA2-pits but not with clathrin-, Cav1-, or Flot1-pits (fig. S7, G and H). Inhibitor experiments revealed that antibody-induced NCAM1 endocytosis was inhibited only by dyngo-4a, with no effect observed from CHC KD, nystatin, or EIPA (fig. S7, I and J). These results indicate that NCAM1 endocytosis occurs, at least partially, through endophilin-mediated endocytosis (i.e., FEME) in neurons. To assess the effect of MPS disruption, we compared the time course of NCAM1 endocytosis, specifically only for the fraction of NCAM1 puncta colocalized with endogenous EndoA2-pits, in WT and βII-spectrin KD neurons (Fig. 4E). Since βII-spectrin KD neurons exhibited ~10% higher NCAM1 surface expression (fig. S7, K and L), we selected neurite regions from both groups with comparable NCAM1 surface levels for our time course analysis. Our results showed that the internalization of the NCAM1 pool that is mediated by EndoA2 was increased in both axonal and dendritic compartments of βII-spectrin KD neurons compared to WT neurons (Fig. 4F), demonstrating that the MPS also restricts ligand-induced FEME. Together, our findings demonstrate that the MPS serves as a regulatory barrier that inhibits both clathrin-dependent and clathrin-independent ligand-induced endocytosis in axonal and somatodendritic compartments.

### The MPS acts as a dynamic barrier modulated by endocytosis and ERK signaling through a positive feedback mechanism

**Fig. 5.**
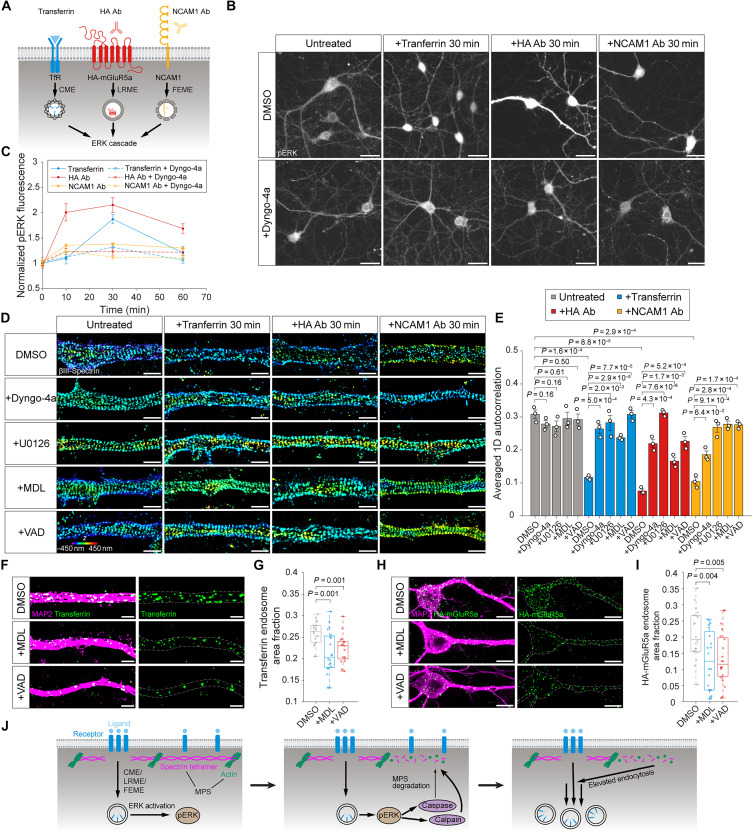
The MPS acts as a dynamic barrier modulated by endocytosis and ERK signaling through a positive feedback mechanism. (**A**) Schematic illustrating ligand-induced ERK activation via three major endocytic pathways: CME of TfR, LRME of HA-mGluR5a, and FEME of NCAM1. (**B**) Top: Epi-fluorescence images showing pERK immunostaining in neurons without ligand treatment, neurons treated with CF568-transferrin, and neurons overexpressing HA-mGluR5a treated with anti-HA antibody. Bottom: The same as the top but with neurons pretreated with dyngo-4a before ligand treatment. Scale bars, 25 μm. (**C**) Time course of ERK activation in neurons under the same conditions as in (B). (**D**) 3D STORM images of immunostained βIII-spectrin in dendrites of neurons under various treatments. First column: neurons pretreated with dimethyl sulfoxide (DMSO), dyngo-4a, U0126, MDL, or VAD. Second column: neurons pretreated with the same inhibitors followed by CF568-transferrin treatment. Third column: neurons overexpressing HA-mGluR5a pretreated with the same inhibitors followed by the anti-HA antibody treatment. Fourth column: neurons pretreated with the same inhibitors followed by anti-NCAM1 antibody treatment. Scale bars, 1 μm. Color scale bar represents the *z*-coordinate information. (**E**) Averaged 1D autocorrelation amplitudes of βIII-spectrin, calculated for the same conditions as in (D). (**F**) SIM images of MAP2 (magenta) and internalized CF568-transferrin (green) in neurons pretreated with DMSO, MDL, or VAD followed by CF568-transferrin treatment. Scale bars, 2 μm. (**G**) Boxplots of transferrin-positive endosome area fractions. (**H**) Confocal fluorescence images of MAP2 (magenta) and internalized HA-mGluR5a (green) in neurons overexpressing HA-mGluR5a pretreated with DMSO, MDL, or VAD followed by anti-HA antibody treatment. Scale bars, 10 μm. (**I**) Boxplots of HA-mGluR5a endosome area fractions. (**J**) Schematic summarizing the proposed positive feedback mechanism: Receptor endocytosis via CME, LRME, or FEME activates ERK signaling, which triggers calpain- and caspase-mediated MPS degradation; MPS disruption in turn facilitates further endocytosis, establishing a positive feedback loop.

We next investigated whether ligand-induced endocytosis can, in turn, affect the MPS. Previously, we demonstrated that ligand binding to the extracellular domain of cannabinoid type 1 receptor (CB1) or NCAM1 induces the activation of the ERK (*23*), a key cell signaling pathway that involved in neuronal development and stress response, and hippocampal learning and survival (*44*). While we observed that CB1 ligand-induced ERK activation resulted in calpain-dependent MPS degradation (*23*), it remains unclear whether this phenomenon is common across other ligand-receptor systems (Fig. 5A). Using an IF-based ERK activation assay previously developed to monitor CB1- and NCAM1-induced ERK activation (*23*), we quantitatively assessed phosphorylated ERK (pERK) levels at different time points after ligand stimulation of three receptors: TfR, HA-mGluR5a, and NCAM1. All ligand treatments induced ERK activation, which persisted for up to 60 min posttreatment (Fig. 5, B and C). Inhibiting ligand-induced TfR, HA-mGluR5a, and NCAM1 endocytosis using dyngo-4a significantly reduced ERK activation (Fig. 5, B and C), indicating that a substantial portion of ERK activation occurs at internalized endosomes and that ligand-induced endocytosis of TfR, HA-mGluR5a, and NCAM1 effectively contributes to ERK signaling. Notably, this contrasts with CB1 ligand–induced ERK activation, which does not rely on CB1 endocytosis and instead appears to occur primarily at the cell surface rather than in endosomes (*23*).

We next investigated whether ligand-induced endocytosis and ERK activation leads to protease-dependent MPS degradation. Using average 1D autocorrelation amplitude analysis, a quantification method we previously developed to measure the integrity of MPS (*23*), we examined the effects of ligand-induced TfR, HA-mGluR5a, or NCAM1 endocytosis on MPS structure in neurons. Quantitative analysis of 3D STROM images of βIII-spectrin revealed significant MPS degradation following ligand stimulation, as evidenced by decreased average 1D autocorrelation amplitudes (Fig. 5, D and E). Inhibiting ligand-induced TfR, HA-mGluR5a, or NCAM1 endocytosis using dyngo-4a prevented MPS degradation (Fig. 5, D and E). Preincubation with U0126, an inhibitor of mitogen-activated protein kinase (MEK), the kinase upstream of ERK, also protected the MPS from degradation (Fig. 5, D and E). These results indicate that both endocytosis and ERK activation are upstream events required for MPS degradation. To identify the proteases involved, we tested the effects of MDL-28170 (MDL, a calpain inhibitor) and Z-VAD-FMK (VAD, a caspase inhibitor). Both of the proteases are known to cleave brain spectrin (*45*) and can be activated by ERK (*46*, *47*). Treatment of neurons with either protease inhibitor rescued MPS integrity, with varying degrees of protection, suggesting that endocytosis-induced MPS degradation is calpain- and caspase-dependent (Fig. 5, D and E).

To further confirm protease-mediated MPS degradation, we examined the characteristic proteolytic profiles of αII- and βII-spectrin breakdown products using Western blotting. Previous studies have well documented that calpain- and caspase-mediated spectrin cleavage yield distinct proteolytic fragments detectable by Western blot: For αII-spectrin, calpain generates a major 150-kDa fragment, whereas caspase produces a 120-kDa fragment; for βII-spectrin, calpain generates 110- and 85-kDa fragments, whereas caspase produces 108- and 80-kDa fragments (*45*). Following ligand-induced endocytosis of TfR, NCAM1, or HA-mGluR5a, the 280-kDa full-length αII-spectrin was primarily cleaved into the 150-kDa fragment, with only a faint 120-kDa fragment observed (fig. S8, A and B), indicating that calpain is the primary protease responsible for endocytosis-induced MPS degradation. Pretreating neurons with MDL markedly reduced the 150-kDa band, confirming its calpain specificity. Pretreatment with VAD modestly attenuated the 150-kDa band, likely reflecting VAD’s known partial inhibition of calpain (*48*). For the 260-kDa full-length βII-spectrin, analysis of its breakdown products yielded results consistent with αII-spectrin, although the calpain-derived (110/85 kDa) and caspase-derived (108/80 kDa) fragments could not be clearly distinguished because of their close molecular weights (fig. S8, A and B). Collectively, these results indicate that ligand-induced MPS degradation occurs primarily via calpain-mediated spectrin cleavage, with minimal contribution from caspase activity.

To further probe whether increased endocytosis alone could degrade the MPS, we investigated whether moderate overexpression of GFP-tagged endocytic pit proteins (i.e., Cav1, Flot1, and EndoA2), which may increase endocytic pit density, could similarly affect MPS integrity. We first confirmed that overexpressing these endocytic pit proteins in neurons significantly increased the endocytic pit area fractions compared to WT neurons, as quantified from the 3D STORM images described above (fig. S9A). We then assessed MPS integrity using epi-fluorescence and STORM imaging. Compared to WT neurons and neurons overexpressing a plasma membrane–associated protein BSAP1 (an endocytic pit-irrelevant control), neurons overexpressing GFP-tagged Cav1, Flot1, or EndoA2 exhibited a moderate decrease in both the average βII-spectrin IF intensity (quantified by epi-fluorescence imaging) and the average 1D autocorrelation amplitude (quantified by STORM imaging) in axons and dendrites (fig. S9, B to E). These findings suggest that increased endocytic pit density and enhanced endocytosis activity alone can induce moderate MPS degradation, further supporting our model that endocytosis contributes to MPS disruption.

So far, our data demonstrate that the MPS suppresses endocytosis, and, conversely, endocytosis can lead to MPS degradation. This reciprocal relationship suggests that endocytosis-induced MPS degradation may be a programmed cellular mechanism designed to rapidly remove the MPS barrier, facilitating accelerated endocytosis once triggered. To further test this hypothesis, we examined whether preventing MPS degradation could suppress ligand-induced endocytosis. For both ligand-induced TfR and HA-mGluR5a endocytosis, we quantified the internalized endocytic pit area fractions following ligand treatment, with or without calpain or caspase inhibitor treatment. Neurons were treated with either protease inhibitor, both before ligand addition and continuously throughout the endocytosis process. Compared to untreated conditions, inhibiting MPS degradation via calpain or caspase inhibitors significantly reduced ligand-induced TfR and HA-mGluR5a endocytosis (Fig. 5, F to I). These results support our model that the MPS acts as a dynamic physical barrier to suppress endocytosis. When rapid endocytosis is required, cells activate ERK-protease pathways to induce calpain- and caspase-dependent MPS degradation, thereby removing the MPS barrier and promoting faster receptor internalization. This mechanism establishes a previously unknown positive feedback loop, wherein endocytosis drives MPS degradation, which in turn accelerates subsequent endocytosis and amplifies downstream signaling to support rapid neuronal responses (Fig. 5J).

### The MPS serves as a neuroprotective barrier by restricting APP endocytosis and Aβ42 accumulation

**Fig. 6.**
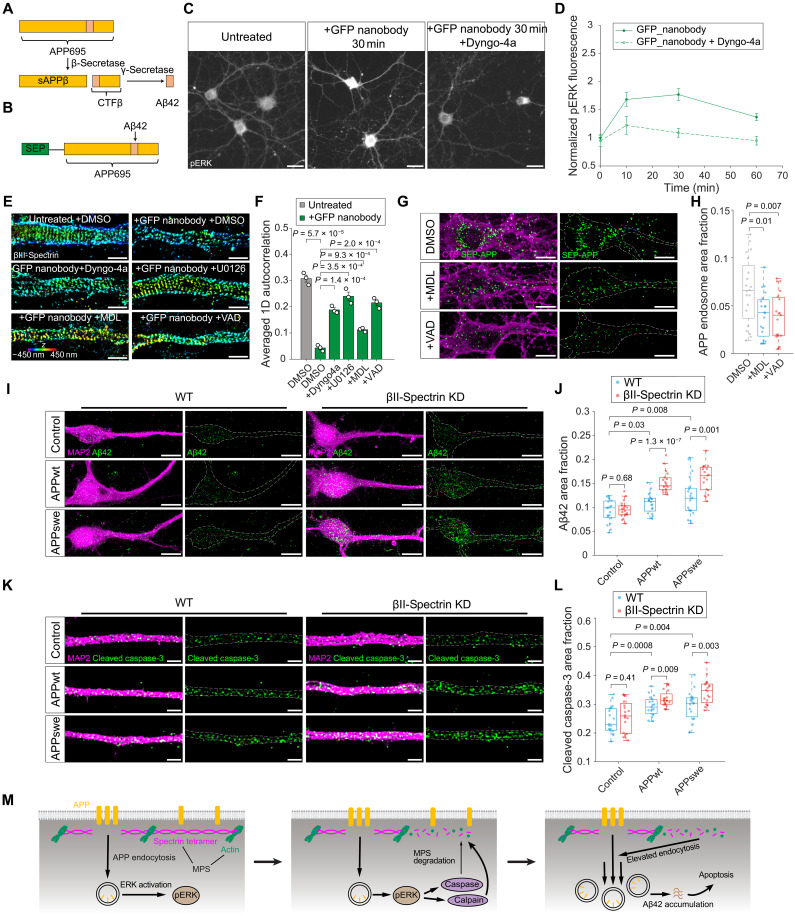
The MPS serves as a neuroprotective barrier by restricting APP endocytosis and Aβ42 accumulation. (**A**) Schematic illustrating the sequential cleavage of APP695 by β-secretase and γ-secretase to produce Aβ42. (**B**) Schematic illustrating the structure of SEP-APP. (**C**) Left: Epi-fluorescence images of pERK in neurons overexpressing SEP-APP without ligand treatment. Middle: The same as the left but treated with GFP nanobody. Right: The same as the middle but with dyngo-4a preincubation before GFP nanobody treatment. Scale bars, 25 μm. (**D**) Time course of ERK activation in neurons under the same conditions as in (C). (**E**) 3D STORM images of immunostained βIII-spectrin in dendrites of neurons pretreated with DMSO, dyngo-4a, U0126, MDL, or VAD followed by GFP nanobody treatment. Scale bars, 1 μm. (**F**) Averaged 1D autocorrelation amplitude of βIII-spectrin, calculated for the same conditions as in (E). (**G**) Confocal fluorescence images of CTB (magenta) and internalized SEP-APP (green) in neurons pretreated with DMSO, MDL, or VAD followed by GFP nanobody treatment. Scale bars, 10 μm. (**H**) Boxplots of SEP-APP endosome area fractions. (**I**) Left: Confocal fluorescence images of MAP2 (magenta) and intracellular Aβ42 (green) in WT neurons, neurons overexpressing APPwt, and neurons overexpressing APPswe. Right: The same as the left but in βII-spectrin KD neurons. Scale bars, 10 μm. (**J**) Boxplots of intracellular Aβ42 area fractions in somatodendritic regions of neurons. (**K**) Left, SIM images of MAP2 (magenta) and cleaved caspase-3 (green) in WT neurons, neurons overexpressing APPwt, and neurons overexpressing APPswe. Right: The same as the left but in βII-spectrin KD neurons. Scale bars, 2 μm. (**L**) Boxplots of cleaved caspase-3 area fractions in dendrites of neurons. (**M**) Schematic illustrating APP endocytosis triggers downstream ERK signaling, leading to MPS degradation through caspase- and calpain-mediated spectrin cleavage. This degradation further accelerates APP endocytosis, promoting intracellular Aβ42 accumulation and caspase-3 activation.

Increasing evidence indicates that dysregulated endocytosis in neurons contributes to the pathogenesis of AD, a progressive neurodegenerative disorder where dementia symptoms gradually worsen over a number of years (*3*, *4*). Elevated production and accumulation of misfolded amyloid-β (Aβ) peptide in the brain correlates with neuronal aging and the AD development. The pathogenic form of Aβ (Aβ42) is generated from APP after cell surface–localized APP undergoes endocytosis and is processed by sequential β- and γ-secretase cleavage in neuronal endosomes and lysosomes (Fig. 6A) (*49*). This raises the possibility that the MPS may have a neuroprotective role by suppressing APP endocytosis, thereby limiting Aβ production and accumulation.

To test this hypothesis, we used a previously established ligand-induced APP endocytosis system (*50*) to manipulate and monitor APP endocytosis in neurons. In this system, a GFP variant, superecliptic pHluorin (SEP), was fused to the N terminus of APP695, the predominant APP isoform in neuronal tissues (Fig. 6B). Treating neurons overexpressing SEP-APP with a fluorophore-conjugated nanobody (~15 kDa) against SEP induced SEP-APP endocytosis in the somatodendritic compartments (*50*). Pretreating neurons with our four described inhibitors revealed that SEP-APP endocytosis involves complex pathways, proceeding through both clathrin-dependent and clathrin-independent pathways (fig. S10, A and B). This finding contrasts with a previous study that identified nanobody-induced SEP-APP endocytosis as strictly clathrin-independent (*50*), potentially due to differences in experimental conditions. We then compared SEP-APP endocytosis rates in both WT and βII-spectrin KD neurons. SEP-APP uptake was significantly faster in βII-spectrin KD neurons (τ = 14.52 ± 0.38 min) compared to WT neurons (τ = 37.21 ± 5.11 min), suggesting that MPS degradation accelerated APP internalization (fig. S10, C and D). Notably, this endocytosis enhancement was not due to an increased APP surface or total expression upon βII-spectrin KD (fig. S10, E to H). Similar to other ligand-induced endocytosis observed in this study, SEP-APP endocytosis activated sustained downstream ERK signaling, as indicated by increased pERK IF (Fig. 6, C and D). Following the nanobody-induced SEP-APP endocytosis, significant MPS degradation was observed, shown by reduced 1D autocorrelation amplitude of the periodic βIII-spectrin distribution (Fig. 6, E and F). Inhibiting SEP-APP endocytosis using dyng-4a or U0126 prevented MPS degradation, indicating that the MPS degradation is endocytosis and ERK dependent. Moreover, this MPS degradation was partially prevented by calpain or caspase inhibitor treatment, indicating that MPS degradation was mediated by calpain- and caspase-dependent spectrin cleavage (Fig. 6, E and F). Western blot analysis of the proteolytic profiles of αII- and βII-spectrin breakdown products indicated that the primary protease responsible for APP endocytosis–induced MPS degradation is calpain (fig. S8C). Conversely, protecting MPS from degradation using calpain or caspase inhibitor significantly reduced SEP-APP endocytosis in neurons (Fig. 6, G and H), reinforcing the role of the MPS as a regulatory barrier that restricts APP internalization. These findings suggested that APP endocytosis promotes ERK signaling, which facilitates MPS degradation, establishing a positive feedback loop that accelerates APP internalization.

We next examined whether increased APP endocytosis in MPS-disrupted neurons leads to elevated Aβ production, a hallmark of AD. To avoid potential confounding effects from SEP tagging, we quantified the Aβ accumulation in cultured mature neurons with and without overexpressing WT APP695 (APPwt) or APP695 bearing the K670M/N671L Swedish mutation (APPswe), a familial AD-associated variant known to enhance Aβ accumulation. We used a previously developed IF-based Aβ42 quantification assay using a highly specific anti-Aβ42 C-terminal antibody (*51*). Immunostaining of intracellular Aβ42, the key pathogenic form of Aβ, revealed elevated Aβ42 levels in neurons overexpressing either APPwt or APPswe, compared to neurons without APP overexpression (i.e., WT neurons), confirming that overexpressing APPwt and APPswe lead to increased intracellular Aβ42 production (Fig. 6, I and J) (*52*). βII-Spectrin KD neurons overexpressing APPwt or APPswe exhibited significantly higher levels of intracellular Aβ42 accumulation than WT neurons overexpressing APPwt or APPswe (Fig. 6, I and J). Aβ42 levels remained comparable between WT and βII-spectrin KD neurons without APP overexpression, indicating that the observed increase in Aβ42 accumulation requires both MPS disruption and APP overexpression. Together, these findings suggest that the MPS plays a neuroprotective role in limiting Aβ42 accumulation, likely by suppressing APP endocytosis and reducing subsequent secretase-mediated APP cleavage in endosomes and lysosomes of neurons.

Intraneuronal Aβ42 accumulation occurs before the formation of extracellular Aβ plaques and is known to trigger a cascade of pathological events, including proteasome and mitochondrial dysfunction, calcium dysregulation, and synaptic impairment (*53*). Since intraneuronal Aβ42 accumulation has been previously linked to activation of caspase-3, a canonical marker of apoptosis, as well as neuronal apoptosis (*52*, *54*), we investigated whether MPS disruption exacerbates Aβ42-mediated neurotoxicity by comparing caspase-3 activations levels in WT and βII-spectrin KD neurons with and without APPwt or APPswe overexpression. Immunostaining for cleaved (activated) caspase-3 using a previously reported, highly specific anti–caspase-3 antibody (*29*) revealed significantly higher caspase-3 activation in neurons overexpressing APPwt or APPswe compared to nonoverexpressing controls, with even greater caspase-3 activation observed in βII-spectrin KD neurons (Fig. 6, K and L), supporting the hypothesis that MPS disruption accelerates APP internalization and subsequent Aβ42 production, thereby promoting neurotoxicity and neuronal apoptosis. The observed patterns of caspase-3 activation mirrored the corresponding Aβ42 accumulation levels across WT and βII-spectrin KD neurons with and without APP overexpression, suggesting that elevated apoptosis signals are likely a direct consequence of increased Aβ42 accumulation. Together, these findings support a model in which the MPS acts as a neuroprotective barrier that restricts APP endocytosis, thereby limiting intraneuronal Aβ42 production, accumulation, and subsequent neuronal apoptosis (Fig. 6M). When the MPS degradation occurs, previously observed in both aged and the neurodegenerative conditions (*55*), APP endocytosis accelerates, leading to pathological Aβ42 accumulation, neuronal dysfunction, and ultimately neuronal death. This mechanism provides insight into how MPS degradation may contribute to the progression of AD and highlights preserving MPS integrity as a potential therapeutic strategy to mitigate APP-related neurodegeneration.

## DISCUSSION

In this study, we leveraged super-resolution fluorescence imaging to visualize the structural interplay between the MPS and endocytic events at nanoscale resolution and identified endocytic clearing structures analogous to those previously reported for CCPs at the AIS and proximal axon (*30*). Notably, we extended these observations to distal axonal and somatodendritic compartments, which were not reported in the initial study (*30*). The absence of such structures in the initial study may be due to the use of immature neurons, in which the somatodendritic MPS had not yet developed into a sufficiently dense and continuous lattice network (*18*). We found that these clearing structures are not unique to CME but also observed for other major endocytic pathways, including LRME, such as caveolin- and flotillin-mediated internalization, as well as FEME. These findings reveal that MPS-based clearings represent a conserved structural feature across multiple endocytic mechanisms. Our super-resolution imaging thus provides direct structural evidence that the MPS functions as a spatial gatekeeper of endocytosis, regulating access through discrete clearings embedded within the periodic actin-spectrin lattice.

Aligned with our structural findings, functional assays comparing the endocytosis rates in neurons with and without MPS disruption support the role of the MPS as a universal regulator of all major endocytic pathways, including CME, LRME, and FEME. Disruption of the MPS not only resulted in a pronounced elevation of the intrinsic, basal-level endocytic activity across multiple pathways but also markedly accelerated ligand-induced internalization events mediated through CME, LRME, and FEME, underscoring the MPS’s central role as a structural suppressor of both constitutive and stimulus-evoked endocytic processes. Our data reveal that ligand-induced endocytosis itself activates a positive feedback loop that further accelerates endocytic uptake: Ligand-induced endocytosis triggers sustained ERK activation, which in turn activates calpain and caspase to cleave spectrin and degrade the MPS. This degradation process effectively removes the MPS-imposed barrier, thereby facilitating faster and more robust endocytic uptake. The presence of this positive feedback loop illustrates a finely tuned mechanism whereby neurons can rapidly enhance endocytosis when rapid signaling or cargo internalization is required. This dynamic interplay underscores the MPS as both a gatekeeper and a responsive element in endocytic regulation.

In addition to CME, our findings reveal that other major endocytic pathways, including LRME and FEME, also occur at the AIS, distal axon, and somatodendritic compartments of neurons under basal (unstimulated) conditions. This broad spatial distribution underscores the pervasive and compartmentally integrated nature of endocytosis in mature neurons, extending beyond the traditionally emphasized role of CME. By using a panel of well-characterized ligands and assessing their colocalization with pathway-specific endocytic structures, we were able to selectively probe the activity of distinct endocytic mechanisms: transferrin and LDL for CME, mGluR5a for LRME, and NCAM1 for FEME. These assays provide a robust framework for the investigation of specific endocytic pathways in neurons.

Our findings also reveal important implications for neuronal health and disease. We observed that MPS degradation accelerates APP endocytosis, leading to increased production and intracellular accumulation of Aβ42, a pathogenic hallmark of AD. The elevated intraneural Aβ42 levels resulting from increased APP internalization were accompanied by downstream neurotoxic events, such as increased activation of caspase-3, which has been observed in Alzheimer’s brain (*56*). Stabilizing the MPS using calpain or caspase inhibitors reduced APP endocytosis, suggesting that the MPS serves as a neuroprotective structure that restricts excessive APP internalization and potentially slows down subsequent Aβ42 generation and caspase-3 activation. Moreover, the positive feedback loop we identified—whereby endocytosis triggers ERK activation, leading to protease-mediated MPS degradation—provides a plausible mechanism through which neurodegeneration may be exacerbated. Supporting this idea, spectrin cleavage products have been proposed as biomarkers for aging and neurodegenerative diseases (*57*). In aging neurons or those subjected to chronic stress, MPS instability may initiate a self-perpetuating cycle of heightened endocytosis and sustained ERK signaling, further accelerating APP internalization and Aβ42 accumulation. This insight is consistent with growing evidence that dysregulated endocytosis and protease activation contribute to Alzheimer’s pathogenesis.

Together, these findings position the MPS as a central dynamic structural regulator of neuronal endocytosis, capable of both constraining and dynamically modulating membrane trafficking across diverse cellular contexts. By uncovering its roles in both physiological regulation and disease-relevant pathways, our study not only expands the conceptual framework of endocytic control in neurons but also highlights the MPS as a potential therapeutic target for modulating endocytosis in neurodegenerative disorders.

## MATERIALS AND METHODS

### Primary culture of mouse hippocampal neurons

All experimental procedures were conducted in accordance with the Guide for the Care and Use of Laboratory Animals of the National Institutes of Health. The protocol (PROTO202101857) was approved by the Institutional Animal Care and Use Committee of the Pennsylvania State University. Primary cultures of hippocampal neurons were prepared as previously described (*18*). Briefly, timed-pregnant CD-1 IGS mice (Charles River Laboratories, Wilmington, MA; 022) were euthanized, and hippocampi were isolated from E18 mouse embryos. The dissected tissues were enzymatically dissociated with 0.25% trypsin-EDTA (1×) (Sigma-Aldrich, T4549) at 37°C for 15 min. Following digestion, the hippocampal tissues were washed three times with Hanks’ balanced salt solution (Thermo Fisher Scientific, 14175095) and then transferred to NbActiv1 culture medium (Transnetyx, NB1500 ml), a premixed formulation comprising Neurobasal, B27, and Glutamax, with the addition of Primocin (100 μg/ml; InvivoGen, ant-pm-2). The tissues were gently triturated in the culture medium until a single-cell suspension was achieved, ensuring that no tissue clumps remained. Dissociated cells were then counted and plated onto poly-d-lysine–coated 18-mm coverslips (Neuvitro, GG-18-1.5-PDL). Cultures were maintained in a humidified incubator at 37°C with 5% CO_2_. Half of the medium volume was replaced every 5 days to maintain optimal culture conditions. Unless otherwise specified, the neurons were fixed between 21 and 28 DIV for subsequent experiments described in this study.

### Antibodies

The following primary antibodies were used in this study: guinea pig anti-tau antibody 1:500 dilution for IF (Synaptic Systems, 314004), mouse anti-tau antibody 1:500 dilution for IF (BD Biosciences, 556319), guinea pig anti-MAP2 antibody 1:500 dilution for IF (Synaptic Systems, 188004), rabbit anti-MAP2 antibody 1:500 dilution for IF (Synaptic Systems, 188002), chicken anti-neurofascin antibody (R&D system, AF3235), rabbit anti-CHC antibody 1:500 dilution for IF (Abcam, ab21679), rabbit anti-Cav1 antibody 1:400 dilution for IF (Cell Signaling Technology, 3238S), mouse anti-Flot1 antibody 1:100 dilution for IF (BD Biosciences, 610820), mouse anti-endophilinA2 antibody 1:100 dilution for IF (Santa Cruz Biotechnology, sc-365704), mouse anti–αII-spectrin (EnCor Biotechnology, MCA-3D7), mouse anti-βII spectrin antibody 1:200 dilution for IF (Santa Cruz Biotechnology, sc-515592), mouse anti-βII spectrin antibody 1:200 dilution for IF (BD Biosciences, 612563), rabbit anti-adducin antibody 1:500 dilution for IF (Abcam, ab51130), chicken anti-GFP antibody 1:500 dilution for IF (Thermo Fisher Scientific, A10262), rabbit anti-GFP antibody 1:500 dilution for IF (Thermo Fisher Scientific, A11122), goat anti-βIII spectrin antibody 1:100 dilution for IF (Santa Cruz Biotechnology, sc-9660), mouse anti-βIII spectrin antibody 1:100 dilution for IF (Santa Cruz Biotechnology, sc-515737), mouse anti-HA antibody 1:200 dilution for HA-mGluR5a internalization and IF (Thermo Fisher Scientific, 26183), rat anti-NCAM1 (CD56) antibody 1:40 dilution for NCAM1 internalization and IF (Cedarlane, CL10008AP), rat anti-TfR (CD71) antibody 1:500 dilution for IF (Bio-Rad, MCA1033GA), goat anti–LDL receptor (LDLR) antibody 1:100 dilution for IF (Thermo Fisher Scientific, PA5-46987), rabbit anti–phospho-ERK antibody 1:300 dilution (Cell Signaling Technology, 4370S), mouse anti-Aβ42 antibody 1:200 dilution for IF (BioLegend, 805501), and rabbit anti–cleaved caspase-3 (Asp175) antibody 1:400 dilution for IF (Cell Signaling Technology, 9661).

The following secondary antibodies were used in this study: Alexa Fluor 647–conjugated donkey anti–guinea pig immunoglobulin G (IgG) antibody 1:800 for IF (Jackson ImmunoResearch, 706-605-148), Alexa Fluor 647–conjugated donkey anti–rabbit IgG antibody 1:800 for IF (Jackson ImmunoResearch, 711-605-152), Alexa Fluor 647–conjugated donkey anti–mouse IgG antibody 1:800 for IF (Jackson ImmunoResearch, 715-605-151), Alexa Fluor 647–conjugated donkey anti–rat IgG antibody 1:800 for IF (Jackson ImmunoResearch, 712-165-153), Alexa Fluor 647–conjugated donkey anti–goat IgG antibody 1:800 for IF (Jackson ImmunoResearch, 705-605-147), Cy3-conjugated donkey anti–rabbit IgG antibody 1:800 for IF (Jackson ImmunoResearch, 711-165-152), Cy3-conjugated donkey anti–mouse IgG antibody 1:800 for IF (Jackson ImmunoResearch, 715-165-151), Cy3-conjugated donkey anti–guinea pig IgG antibody 1:800 for IF (Jackson ImmunoResearch, 706-165-148), Cy3-conjugated donkey anti–rat IgG antibody 1:800 for IF (Jackson ImmunoResearch, 712-165-153), Alexa Fluor 488–conjugated donkey anti–mouse IgG antibody 1:800 for IF (Jackson ImmunoResearch, 715-545-150), Alexa Fluor 488–conjugated donkey anti–guinea pig IgG antibody 1:800 for IF (Jackson ImmunoResearch, 706-545-148), Alexa Fluor 488–conjugated donkey anti–rabbit IgG antibody 1:800 for IF (Jackson ImmunoResearch, 711-545-152), donkey anti–mouse IgG antibody (Jackson ImmunoResearch, 715-005-151), donkey anti–rat IgG antibody (Jackson ImmunoResearch, 712-005-153), donkey anti–rabbit IgG antibody (Jackson ImmunoResearch, 711-005-152), and donkey anti–chicken IgG antibody (Jackson ImmunoResearch, 703-005-155) CF583R-conjugated secondary antibodies were made by conjugating the unlabeled secondary antibodies with CF583R succinimidyl ester (Biotium, 96084) to achieve a labeling efficiency of ~2 dyes per antibody, as previously described (*58*).

### Lentivirus production and transduction of hippocampal neurons

Plasmids and gene segments used in this study were purchased from Addgene, GeneCopoeia, and Twist Bioscience and subsequently cloned into the lentiviral expression vector FUGW (Addgene, plasmid #14883), through Gibson assembly reaction (New England Biolabs, E5510S) according to the manufacturer’s instructions. Coding sequences for CAV1, FLOT1, SH3GL1 (synthesized by Twist Bioscience), APP695 (Addgene, plasmid #114193), and Basp1 (GenoCopoeia, plasmid # EX-Mm10271-M98) were fused to a C-terminal GFP via a flexible polypeptide linker and cloned into the FUGW vector, generating GFP-tagged constructs for Cav1, Flot1, EndoA2, APPwt, and Basp1. Two point mutations (K670N and M671L) were introduced into APPwt-GFP to generate the familial AD mutant APPswe-GFP construct. The coding sequence of Grm5a (Addgene, plasmid #197305) was cloned into FUGW vector, with an HA tag inserted between Ser^22^ and Ser^23^ codons of Grm5a, as previously described (*43*), to generate HA-mGluR5a construct. The coding sequence of APP695 was cloned into FUGW vector, with SEP and BBS tags inserted, as previously described (*59*), to generate SEP-APP construct.

Lentiviruses were produced by cotransfecting Lenti-X 293T cells with 6 μg of lentiviral expression vector encoding the gene of interest, 4.5 μg of Δ8.9 vector (*60*) (gift from D. Baltimore, California Institute of Technology), and 3 μg of VSVG packaging vector (Addgene, plasmid #8454) in a 10-cm petri dish using Lipofectamine 3000 transfection reagent (Thermo Fisher Scientific, L3000008). One day before transfection, Lenti-X 293T cells were seeded in Dulbecco’s modified Eagle’s medium to reach ~80% confluency. One hour before transfection, the medium was replaced with viral packaging medium, which is Opti-MEM supplemented with 5% fetal bovine serum and 200 μM sodium pyruvate. The first batch of viral supernatant was collected 24 hours posttransfection and stored at 4°C, and the medium was replaced with fresh packaging medium added to the cells. A second batch of supernatant was harvested 48 hours posttransfection, combined with the first batch, centrifuged at 1500*g* for 10 min, filtered through a 0.45-μm pore-size membrane (VWR Inc., 76479-020), and concentrated using the Lenti-X concentrator (Clontech, 631231) at 4°C overnight. The viral titers were determined before the samples were snap-frozen in liquid nitrogen. Lentiviruses were added to neuronal cultures at DIV 4 to 5.

### KD of βII-spectrin or CHC by shRNA adenovirus

The adenovirus-expressing shRNA were purchased from SignaGen Laboratories. The sense sequences of the βII-spectrin shRNA are 5′-GCATGTCACGATGTTACAA-3′ and 5′-GGATGAAATGAAGGTGCTA-3′, as previously described (*61*). The sense sequence of CHC shRNA is 5′-GTTGGTGACCGTTGTTATG-3′, as previously described (*42*). The adenovirus expressing a scramble shRNA sequence was used as a control (Vector BioLabs, 1122). Adenoviruses were added to the neuronal cultures at DIV 2 for βII-spectrin KD and at DIV 7 for CHC KD. The KD efficiencies were confirmed by IF staining.

### Neuron fixation and immunostaining for fluorescence imaging

Cultured hippocampal neurons were fixed using 4% (w/v) paraformaldehyde (PFA) in phosphate-buffered saline (PBS) containing 4% (w/v) sucrose for 20 min at room temperature (RT). Following fixation, neurons were washed three times in PBS, and permeabilized with 0.2% (v/v) Triton X-100 in PBS for 10 min, unless otherwise stated. Neurons were then blocked in the blocking buffer containing 3% (w/v) bovine serum albumin (BSA) in PBS for 1 hour at RT, followed by incubation with primary antibodies diluted in blocking buffer overnight at 4°C. The following day, the neurons were washed three times with PBS and incubated with fluorophore-conjugated secondary antibodies in blocking buffer for 1 hour at RT. After final washes in PBS, the samples were processed for imaging.

For immunostaining of endogenous caveolin, the neurons were first fixed with 4% (w/v) PFA at RT, followed by treatment with cold methanol at −20°C for 10 min. Neurons were then permeabilized with 0.2% (w/v) saponin in PBS for 10 min (*33*), followed by BSA blocking and incubation with primary and secondary antibodies using the protocol described above. For immunostaining of intracellular Aβ42, neurons were fixed with 4% (w/v) PFA and permeabilized with 0.2% (w/v) saponin in PBS for 10 min (*51*), followed by BSA blocking and incubation with primary and secondary antibodies using the protocol described above.

For dual-color staining of βII-spectrin and plasma membrane markers, WGA, or CTB, neurons were fixed with 4% (w/v) PFA in PBS, followed by incubation with AF647-conjugated WGA (5 μg/ml; Thermo Fisher Scientific, W32466) or AF647-conjugated CTB (1 μg/ml; Thermo Fisher Scientific, C34778) for 10 min. The neurons were then postfixed with 4% PFA in PBS for 30 min, permeabilized with 0.2% (w/v) Triton X-100 in PBS for 10 min, and subsequently blocked and immunostained for βII-spectrin using primary and secondary antibodies.

For immunostaining of surface-expressed proteins including TfR, LDLR, HA-mGluR5a, NCAM1, and SEP-APP, the neurons were fixed with 4% PFA in PBS without permeabilization, followed by BSA blocking and incubation with respective primary and secondary antibodies using the protocol described above. For immunostaining of pERK, the neurons were fixed at various time points following ligand-induced ERK activation. The neurons were fixed with 4% PFA, followed by treatment with cold methanol at −20°C for 10 min and permeabilization with 0.2% Triton X-100 for 10 min before BSA blocking and incubation with primary and secondary antibodies according to the manufacturer’s instructions.

### Western blotting of neuronal lysates

Neuronal cultures under each specified condition were lysed in radioimmunoprecipitation assay buffer (Thermo Fisher Scientific, 89900), supplemented with 1× protease inhibitor cocktail (Thermo Fisher Scientific, 87786) and 1× phosphatase inhibitor cocktail (Thermo Fisher Scientific, 78420), following the manufacturer’s instructions. The neuronal lysates were then centrifuged at 14,000*g* for 15 min at 4°C to remove insoluble debris, snap-frozen, and stored at −80°C until use. Protein concentrations of neuronal culture lysates were determined by BCA protein assay kits (Thermo Fisher Scientific, 23227) with albumin standards, following the manufacturer’s instructions. Equal protein amounts (20 μg per lane) were prepared in Laemmli buffer (Bio-Rad, 1610747) and resolved by SDS–polyacrylamide gel electrophoresis on 4 to 20% gradient gels using a tris-glycine running buffer (25 mM tris, 192 mM glycine, and 0.1% SDS). Proteins were then transferred to polyvinylidene difluoride membranes (Bio-Rad, 1620260) using the Trans-Blot Turbo transfer system (25 V, 1 A, 30 min). Membranes were processed with the Fast Western Kit (Thermo Fisher Scientific, 35060) and probed with mouse anti–αII-spectrin (EnCor Biotechnology, MCA-3D7) or mouse anti–βII-spectrin (BD Biosciences, 612563). Molecular weights of intact proteins and their breakdown products were assessed by comparison with molecular weight markers (Bio-Rad, 1610374).

### Ligand-induced endocytosis assays

#### Transferrin and LDL endocytosis

Neuronal culture media were replaced with prewarmed Neurobasal medium (Thermo Fisher Scientific, 21103049) without supplements. Right after medium change, the neurons were treated with CF568-conjugated transferrin (50 μg/ml; Biotium, 00083) or Dil-conjugated LDL (10 μg/ml; Thermo Fisher Scientific, L3482). At the indicated time points, the neurons were quickly washed twice with ice-cold PBS, followed by three washes with ice-cold acid solution [150 mM glycine-HCl (pH 2.2)] (*62*) to remove surface-bound fluorescent ligands and three additional washes with ice-cold PBS. The neurons were then fixed with 4% (w/v) PFA in PBS containing 4% (w/v) sucrose for 20 min at RT. For transferrin uptake analysis, the neurons were permeabilized with 0.2% (v/v) Triton X-100 for 10 min and immunostained with MAP2. For LDL uptake analysis, the neurons were stained with AF647-conjugated CTB (Thermo Fisher Scientific, C34778) without the permeabilization step.

#### HA-mGluR5a endocytosis

Neuronal culture media were replaced with prewarmed Neurobasal medium without supplements. Right after medium change, neurons expressing HA-mGluR5a through lentiviral transduction were treated with mouse anti-HA antibody (5 μg/ml). At the indicated time points, the neurons were quickly washed twice with ice-cold PBS, followed by three washes with ice-cold acid solution, and then three additional washes with ice-cold PBS. The neurons were then fixed with 4% PFA at RT for 20 min and incubated overnight at RT with unconjugated anti-mouse secondary antibody (0.15 mg/ml) to block residual surface-bound anti-HA antibodies. After an additional 4% PFA fixation at RT for 30 min, the neurons were permeabilized with 0.2% (v/v) saponin for 10 min, 3% (w/v) BSA in PBS for 1 hour at RT, and incubated with MAP2 primary antibody overnight at 4°C, followed by the incubation of secondary antibodies at RT for 60 min for MAP2 and HA. For costaining of endogenous caveolin, the neurons were treated with cold methanol at −20°C for 10 min before saponin permeabilization.

#### NCAM1 endocytosis

Neuronal culture media were replaced with prewarmed Neurobasal medium without supplements. Right after medium change, the neurons were treated with rat anti-NCAM1 antibody (5 μg/ml). At the indicated time points, the neurons were quickly washed three times with ice-cold PBS and fixed with 4% PFA for 20 min. To block surface-bound primary antibodies, the neurons were incubated overnight at RT with Cy3-conjugated donkey anti-rat secondary antibody (0.15 mg/ml). After an additional 4% PFA fixation for 30 min, the neurons were permeabilized with 0.2% Triton X-100 for 10 min, blocked with 3% (w/v) BSA in PBS for 1 hour at RT, and incubated with AF647-conjugated donkey anti-rat secondary antibody at RT for 60 min to detect internalized NCAM1 (*63*). For costaining of endogenous EndoA2, the neurons were incubated overnight at RT with unconjugated donkey anti-rat secondary antibody (0.15 mg/ml) to block surface-bound primary antibodies, and neurons were additionally incubated with primary antibody against EndoA2 after Triton X-100 permeabilization, followed by secondary antibody incubation.

#### SEP-APP endocytosis

Neuronal culture media were replaced with prewarmed Neurobasal medium without supplements. Right after medium change, the neurons expressing SEP-APP were treated with GFP nanobody (0.5 μg/ml; ProteinTech, gb2AF647-50). At the indicated time points, the neurons were quickly washed twice with ice-cold PBS, followed by three washes with ice-cold acid solution, and three additional washes with ice-cold PBS. Neurons were then fixed with 4% PFA at RT and stained with AF647-CTB.

### Pharmacological inhibition of endocytosis and protease activity

Neuronal culture media were replaced with prewarmed Neurobasal medium without supplements. To investigate the endocytic pathways involved in ligand-induced internalization, the neurons were pretreated with 15 μM dyngo-4a (Cayman Chemical, 29479), 20 μM nystatin (Dot Scientific, DSN82020-5) or 40 μM EIPA (Cayman Chemical, 14406) for 30 min before ligand addition to investigate the endocytic pathways involved in ligand-induced internalization. To inhibit MEK activity, the neurons were pretreated with 20 μM U0126 (Cell Signaling Technology, 9903S) for 15 min before ligand addition. To inhibit protease activity, the neurons were treated with 50 μM MDL (Santa Cruz Biotechnology, sc-201301) or 50 μM VAD (APExBIO, A1902-1) for 15 min before ligand addition to inhibit the calpain and caspase activities, respectively.

### Wide-field epi-fluorescence imaging

Wide-field epi-fluorescence imaging was performed using a home-built microscope based on an Olympus IX-73 body, equipped with a 20× [numerical aperture (NA) 0.80] UPlanXApo air immersion objective, a 60× (NA 1.4) PlanApo oil immersion objective (Olympus), and a complementary metal-oxide semiconductor (CMOS) camera (Thorlabs, CS126MU). A Lumencor SOLA light engine (SOLA U-nIR) was used as the light source for illumination. The 20× objective was used for imaging βII-spectrin, CHC, and surface receptors to quantify their expression levels. The average fluorescence intensity was quantified from 20 to 25 randomly selected imaging fields, with background fluorescence subtracted and the average fluorescence intensity normalized. The 60× objective was used for imaging LDL internalization, and the average area fraction of cellular area occupied by LDL endosomes was quantified from 20 to 25 randomly selected fields.

### Confocal and SIM imaging

Confocal and SIM imaging were performed using a custom microscope built on an Olympus IX-83 body, equipped with the CrestOptics X-Light V3 spinning disk confocal unit in combination with DeepSIM X-light super-resolution system module. Imaging was performed using either a 60× (NA 1.42) UPlanXApo or a 100× (NA 1.40) UPlanSApo oil immersion objective (Olympus), and an ORCA-Fusion BT digital CMOS camera (Hamamatsu, C15440-20UP). LDI-NIR-7 Laser Diode Illuminator (89 North) was used as the light source for illumination. For confocal imaging, images were acquired using Multi-Dimensional Acquisition module in Micro-Manager, with multicolor-channel acquisition and z-stack collection. AF488 (or GFP), Cy3, and AF647 were sequentially excited. Z-series images were collected from the top to the bottom of neurons, with a step size of 0.2 to 0.5 μm between planes. Final confocal images were reconstructed using maximum intensity z-projection. For SIM imaging, image acquisition and reconstruction were performed with the CrestOptics Super Resolution plugin integrated in Micro-Manager, allowing multicolor-channel SIM imaging. Stitching of SIM images was performed using the “Grid/Collection stitching” plugin in ImageJ.

### STORM imaging

The STORM setup was built on a Nikon Eclipse-Ti2 inverted microscope, equipped with a 100× (NA 1.45) Plan Apo oil-immersion objective (Olympus) and an EM-CCD camera (Andor iXon Life 897). Lasers 405 nm (Coherent, OBIS 405 nm LX, 1265577; 140 mW), 488 nm (Coherent, Sapphire 488-500 LPX CDRH, 1416094; 300 mW), 560 nm (MPB Communications Inc., 2RU-VFL-P-1500-560-B1R; 1500 mW), and 642 nm (MPB Communications Inc., 2RU-VFL-P-2000-642-B1R; 2000 mW) were introduced through the back port of the microscope. The laser beams were shifted toward the edge of the objective to achieve near-total internal reflection illumination, ensuring selective excitation of fluorophores within a few micrometers of the coverslip surface. For single-color 3D STORM imaging, AF647 was imaged. For dual-color STORM imaging, AF647 and CF583R were sequentially imaged as previously described (*58*). A cylindrical lens was inserted into the detection path to introduce ellipticity into the point spread function, allowing determination of the z-positions of single molecules from the ellipticity of their images (*32*). Samples were imaged in an oxygen scavenging imaging buffer, containing 100 mM tris-HCl (pH 7.5), 100 mM cysteamine (Sigma-Aldrich, G8270-100G), 5% (w/v) glucose (Sigma-Aldrich, G8270-100G), glucose oxidase (0.8 mg/ml; Sigma-Aldrich, G2133-10KU), and catalase (40 μg/ml; Sigma-Aldrich, C100-50MG). During imaging, continuous illumination of 642- or 560-nm laser (~2 kW/cm^2^) was used to excite AF647 or CF583R molecules, respectively, switching them into the dark state. Continuous illumination of the 405-nm laser (0 to 1 W/cm^2^) was used to reactivate the fluorophores to the emitting state, and the illumination power was controlled so that at any given time, only a small, optically resolvable fraction of the fluorophores in the field of view were in the emitting state. A typical single-color 3D STORM image was reconstructed from ~30,000 image frames acquired at a frame rate of 110 Hz. For dual-color 3D STORM, ~60,000 frames (30,000 per color-channel) were acquired under the same conditions. The recorded STORM movies were analyzed using established methods (*32*). Super-resolution images were reconstructed from the molecular coordinates by depicting each location as a 2D Gaussian peak. Color scale bar represents the z-coordinate information. For dual-color STORM analysis, each color-channel was processed independently, and the resulting images were aligned using fluorescent microspheres (Thermo Fisher Scientific, F8803) visible in both channels.

### Data quantification and analysis

All subsequent data quantification and analyses were performed using custom MATLAB scripts.

#### Quantification of membrane-localized and internalized endocytic pits

Fifty to one hundred randomly selected axon segments (~3 μm in length) and dendrite segments (~2 μm in length) were analyzed to determine the densities, lateral (*x/y* plane) and radical (*y/z* plane) distributions of endocytic pits in axonal and dendritic compartments, respectively. Single-molecule localizations of the MPS and endocytic pits from 3D STORM images were analyzed using density-based spatial clustering of applications with noise (DBSCAN) algorithm to identify molecular clusters (i.e., endocytic pits) in 3D space. The volume of each endocytic pit was calculated using convex hull estimation. Following molecular cluster identification and removal of noncluster noise, the single-molecule localizations of both MPS and endocytic pits were projected onto the *y/z* plane. Plasma membrane boundaries were delineated by generating *y/z* projections of the MPS localizations, followed by binary segmentation and elliptical fitting to define the plasma membrane contour. To quantitatively assess the subcellular localization of endocytic pits in the *y/z* cross-sectional view of neurites, we classified each endocytic pit as either surface-localized or internalized based on its spatial position relative to the neurite contour. Specifically, we defined two geometric parameters: $d_{1}$, the distance from the centroid of an endocytic pit to the center of the fitted ellipse representing the neurite shape in the *y/z* view, and $d_{2}$, the distance from the fitted ellipse center to its boundary along the line connecting the ellipse center to the pit centroid. The ratio $d_{1}/d_{2}$was then used to determine localization. Endocytic pits with $d_{1}/d_{2}<0.75$ were classified as internalized, while those with $0.75\leq d_{1}/d_{2}\leq 1$ were considered surface localized. Pits falling outside the neurite contour ($d_{1}/d_{2}>1$) were excluded from further analysis.

#### Analysis of spatial segregation between surface-localized endocytic pits and the MPS

Following clustering, single-molecule localizations of both MPS and surface-localized endocytic pits (single-molecule localizations for the internalized endocytic pits were removed) from dual-color 3D STORM images were projected onto the *x/y* plane and subsequently binarized. We used two complementary methods to validate the exclusion pattern between membrane-localized endocytic pits and the MPS. The first method is to quantify and compare PCCs between endocytic pits and the MPS under both experimental and randomized conditions. To assess statistical significance, spatial randomization was performed by repositioning each endocytic pit cluster within the boundary defined by the MPS regions while avoiding overlaps between clusters. This randomization process was repeated 100 times to generate a null distribution of PCC values for the comparison with experimental data.

The alternative method is to generate averaged dual-color STORM images centered on individual endocytic pits. For each endocytic pit, the corresponding image—containing both the pit and its surrounding MPS structure—was aligned such that the centroid of the endocytic pit was placed at the center of a new composite frame. These frames were then merged into a composite image with intensity normalization for visualization. The final composite image was used to generate the radial intensity profiles of both MPS and endocytic pits, using the Radial Profile plugin in ImageJ. Membrane-associated endocytic pits were classified into two categories based on their spatial relationship to the periodic MPS lattice: class I pits that do not overlap with the MPS and class II pits that overlap with the MPS.

#### 1D autocorrelation analysis

For 1D autocorrelation analysis of the periodic MPS lattice in axons and dendrites, single-molecule localizations from STORM images were projected onto the longitudinal axis of randomly selected axon or dendrite segments. 1D autocorrelation functions were calculated from these 1D projected signals. To obtain the average autocorrelation function, the autocorrelation curves from 30 to 50 randomly selected axon segments (~2 μm in length) or dendrite segments (~3 μm in length) were averaged for each condition. The average 1D autocorrelation amplitude was defined as the difference between the first peak (at ~190 nm) and the average of the two first valleys (at ~95 and ~285 nm) in the average 1D autocorrelation curve, which provides a quantitative measure of periodicity of the MPS lattice in neurites, as previously described (*15*).

#### Quantification of endocytic pit area fraction

The endocytic pit area fraction was defined as the ratio of the area occupied by endocytic pits to the area labeled by neuronal compartment markers, including neurofascin, tau, MAP2, CTB, or surface NCAM1. For markers, images were first smoothed and binarized, after which the largest connected boundary was extracted and converted into a binary mask. This mask was then refined using hole filling and morphological closing to ensure accurate delineation of the neuronal compartments. For the endocytic pits, fluorescence signals within these marker-defined regions were processed by adaptive thresholding, followed by morphological opening to enhance puncta detection. Connected components were then filtered on the basis of size criteria, and holes were filled to exclude incomplete detections. The average area fraction was calculated from 20 to 25 randomly selected imaging regions for each experimental condition.

### Statistical analysis

All experiments were independently replicated three times with similar results. In boxplots, the horizontal line represents the median, and the box denotes the interquartile range (IQR), with boundaries indicating the first and third quartiles. Whiskers extend to 1.5 × IQR, with potential outliers omitted. Data in the bar plots and time-course curves are presented as means ± SEM. A two-sided, unpaired Student’s *t* test was used to assess statistical significance between two experimental conditions, and the corresponding *P* values are indicated in the figures. A two-sided, paired Student’s *t* test was used to assess statistical significance between PCCs under experimental and randomized conditions, and the corresponding *P* values are indicated in the figures.
