# Supplementary material for: Membrane-associated periodic skeleton regulates major forms of endocytosis in neurons through a signaling-driven positive feedback loop
Source: Sci Adv. 2026 Feb 11;12(7):eaeb0803. doi: 10.1126/sciadv.aeb0803 (PMC12893284; doi:10.1126/sciadv.aeb0803)
Supplement: Supplementary file 1 — Figs. S1 to S10 [file sciadv.aeb0803_sm.pdf]

Science Advances
AAAS

Supplementary Materials for

Membrane-associated periodic skeleton regulates major forms of endocytosis in neurons through a signaling-driven positive feedback loop

Jinyu Fei *et al.*

Corresponding author: Ruobo Zhou, ruobo.zhou@psu.edu

Sci. Adv. **12**, eaeb0803 (2026)
DOI: 10.1126/sciadv.aeb0803

This PDF file includes:

Figs. S1 to S10

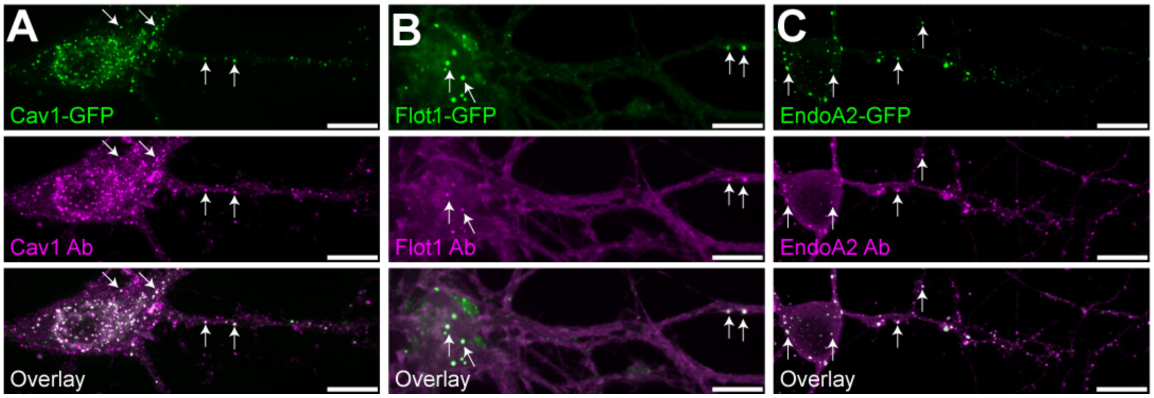

Fig. S1. Validation of antibodies and immunofluorescence-based labeling of caveolin-, flotillin-, and endophilinA2-positive endocytic pits, related to Fig. 1. (**A**) Confocal fluorescence images of neurons exogenously expressing GFP-tagged Cav1 and immunostained for Cav1. Regions of strong colocalization are indicated by white arrows. (**B**) Same as (**A**), but for neurons exogenously overexpressing GFP-tagged Flot1 and immunostained for Flot1. (**C**) Same as (**A**), but for neurons exogenously expressing GFP-tagged EndoA2 and immunostained for EndoA2. Scale bars: 10 µm.

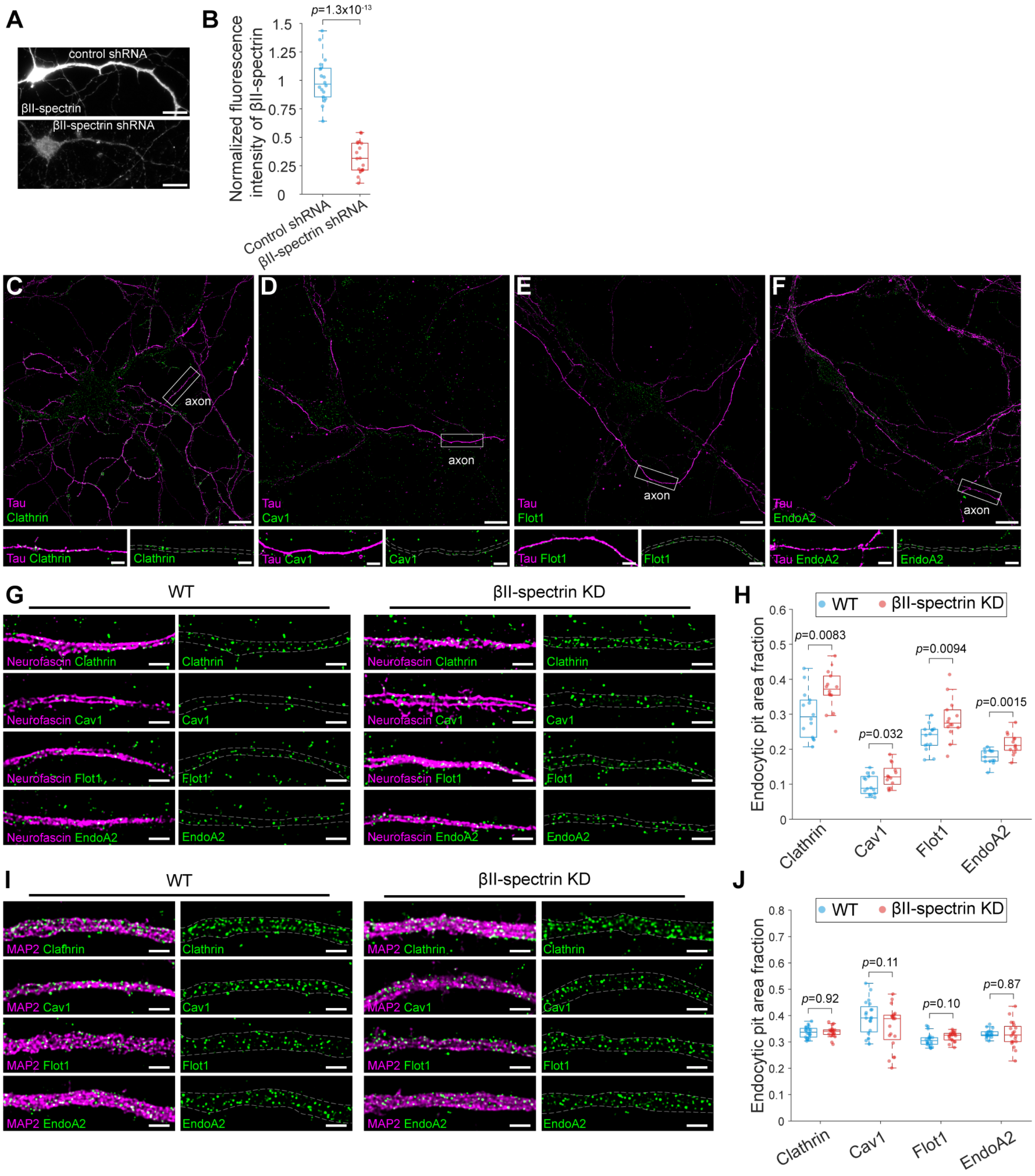

Fig. S2. Basal endocytosis activities in distal axons and the effect of MPS disruption on the basal endocytosis in the Axon Initial Segment (AIS), related to Fig. 1. (**A**) Widefield epi-fluorescence images of βII-spectrin in neurons transduced with adenoviruses expressing either scrambled (control) shRNA or βII-spectrin shRNA. Scale bar: 25 µm. (**B**) Boxplots showing the normalized fluorescence intensity of βII-spectrin. (**C-F**) Stitched SIM images showing the distributions of endogenous endocytic pits, clathrin (**C**), Cav1(**D**), Flot1 (**E**), or EndoA2 (**F**) in wild-type (WT) neurons. Endocytic pits are shown in green, tau, a distal axon marker, is shown in magenta. Scale bar: 10 µm. Bottom: Enlarged SIM images of the boxed

regions on the top, corresponding to distal axon compartments. Scale bar: 2 μm. (**G)** Left: SIM images of neurofascin (magenta) and endogenous endocytic pits (green; clathrin, Cav1, Flot1, or EndoA2) at the AIS of WT neurons. Right: Same as Left, but in βII-spectrin knockdown (KD) neurons. Scale bars: 2 μm. (**H**) Boxplots showing the area fraction of endogenous endocytic pits at the AIS of WT and βII-spectrin KD neurons. (**I)** Left: SIM images of MAP2 (magenta) and endogenous endocytic pits (green) in dendrites of WT neurons (DIV 7). Right: Same as Left, but in βII-spectrin knockdown (KD) neurons (DIV 7). Scale bars: 2 μm. (**J**) Boxplots showing the area fraction of endogenous endocytic pits in dendrites of WT and βII-spectrin KD neurons (DIV 7). Boxplots show the median and boundaries (first and third quartile); Whiskers denote 1.5 times the interquartile range of the box. *p*-values calculated with two-sided unpaired Student's *t*-test.

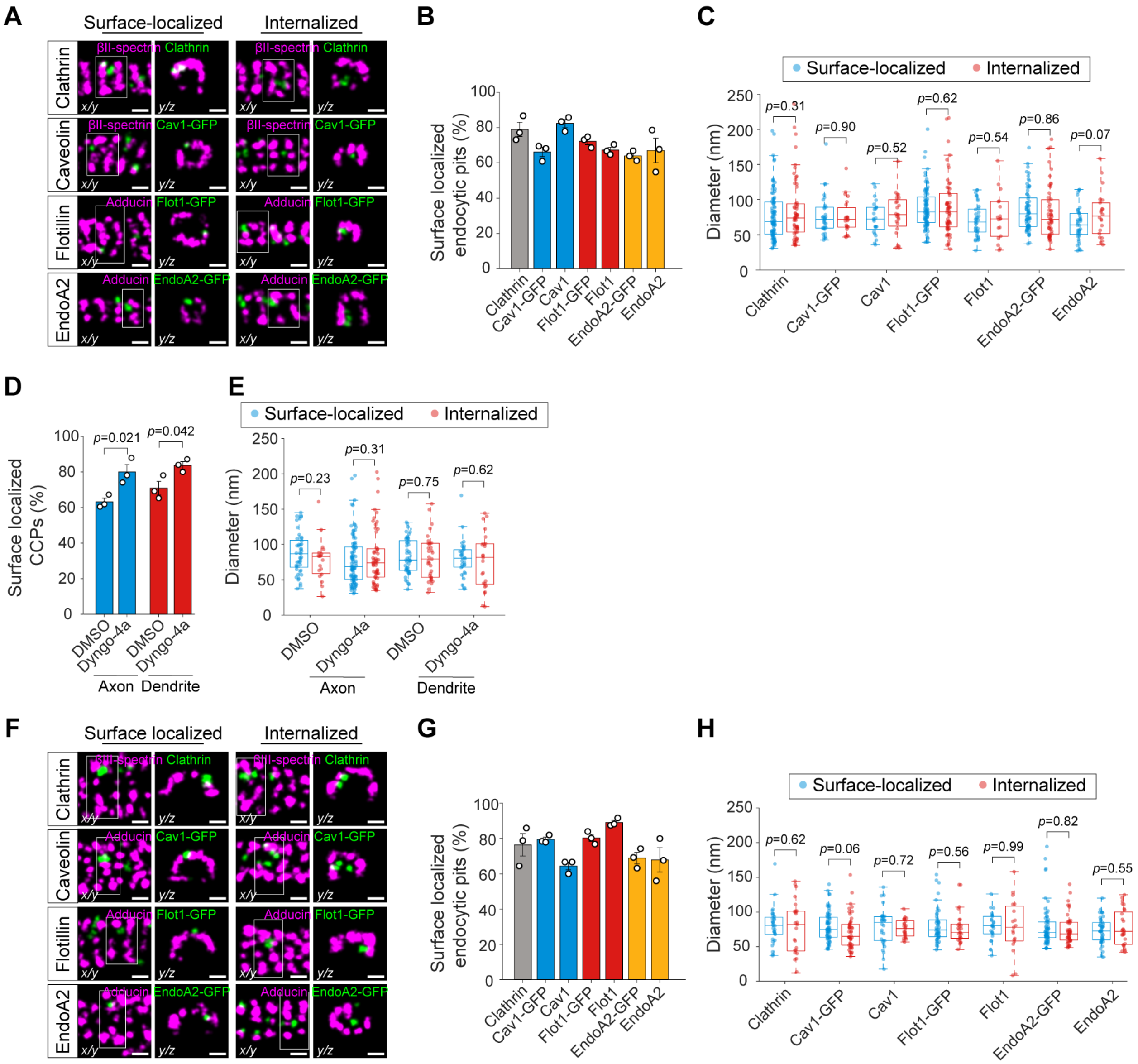

Fig. S3. Quantification of surface-localized and internalized endocytic pits in axons and dendrites, related to Fig. 2 and Fig. 3. (**A**) Left: Representative dual-color STORM images of the MPS (magenta) and surface-localized endocytic pits (green) in axons. Endogenous clathrin and exogenously expressed Cav1 were co-stained with βII-spectrin, while exogenously expressed Flot1 and exogenously expressed EndoA2 were co-stained with adducin. The *y/z* view corresponds to the white-boxed region in the *x/y* view. Right: Same as Left, but for endocytic pits internalized within the axonal cytoplasm. Scale bars: 200 nm. (**B**) Average percentage of surface-localized endocytic pits in axons. Quantification includes only endogenous clathrin, and both endogenous and exogenously expressed Cav1, Flot1, and EndoA2. (**C**) Boxplots showing the diameters of surface-localized and internalized endocytic pits in axons. Quantification includes the same categories as in (**B**). (**D**) Average percentage of surface-localized CCPs in axons and dendrites under DMSO (control) or dyngo-4a treatment. **(E)** Boxplots showing the diameters of membrane-localized and internalized CPPs in axons and dendrites under DMSO or dyngo-4a treatment. **(F-H)** Same as (**A-C**), but for dendrites. Data are presented as mean ± s.e.m. (*n* = 3 biological replicates

per condition). Boxplots show the median and boundaries (first and third quartile); Whiskers denote 1.5 times the interquartile range of the box. *p*-values calculated with two-sided unpaired Student's *t*-test.

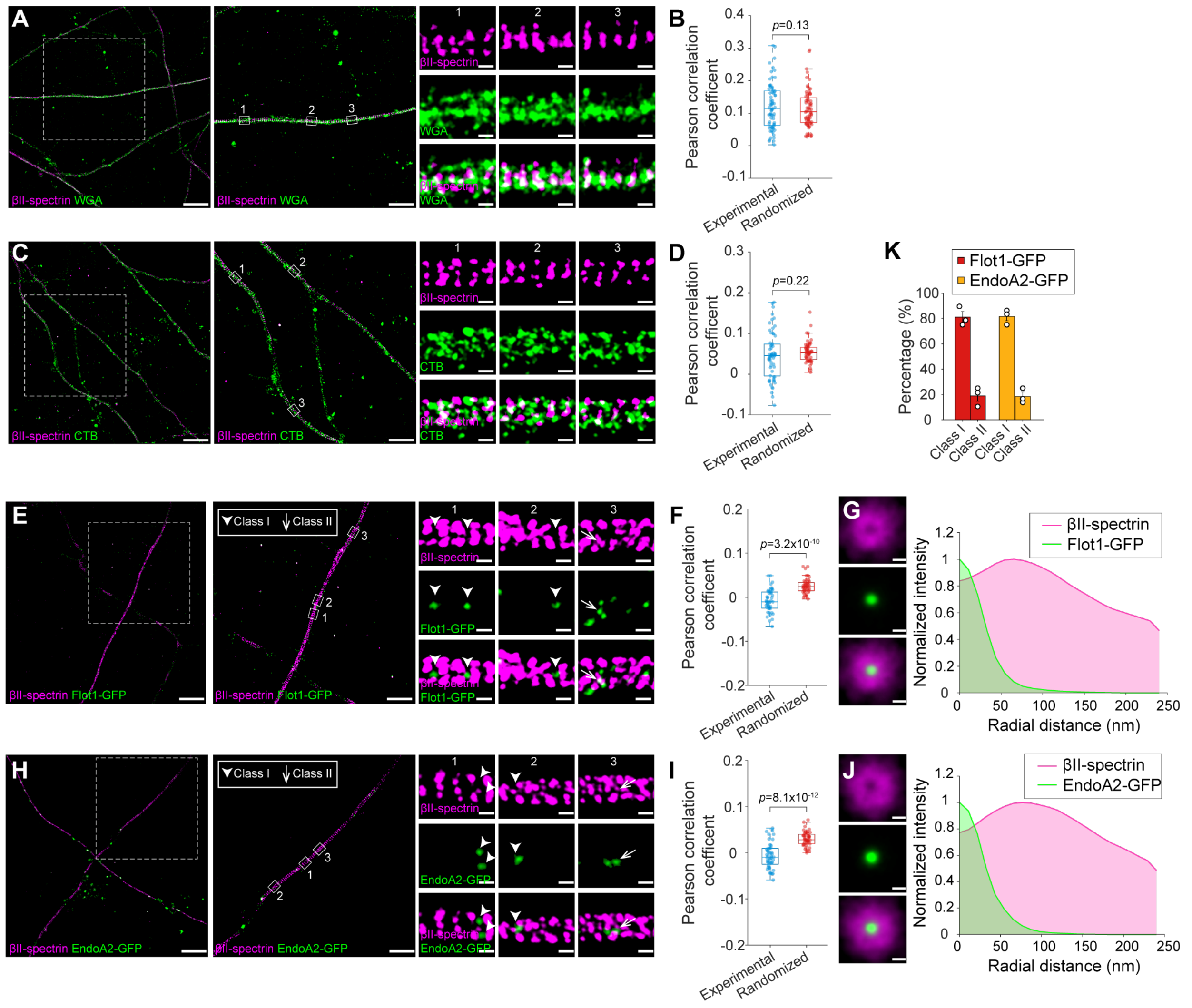

Fig. S4. Endocytic pits are localized within "clearings" of the MPS lattice in axons, related to Fig. 2. (**A**) Dual-color STORM images of βII-spectrin (magenta) and wheat germ agglutinin (WGA, green) in axons. Scale bars: 10 µm (left), 5 µm (middle), 200 nm (right). (**B**) Pearson correlation coefficients between βII-spectrin and WGA under experimental and randomized conditions. (**C, D**) Same as (**A, B**), but for βII-spectrin (magenta) and cholera toxin subunit B (CTB, green). (**E**) Left: Dual-color STORM images of βII-spectrin (magenta) and exogenously expressed Flot1 (green) in axons. Right: Magnified views of Class I and Class II Flot1-pits in the boxed regions. Scale bars: 10 µm (left), 5 µm (middle), 200 nm (right). (**F**) Pearson correlation coefficients between βII-spectrin and exogenously expressed Flot1 under experimental and randomized conditions. (**G**) Left: Averaged dual-color STORM images of βII-spectrin (magenta) and exogenously expressed Flot1 (green), generated by aligning individual STORM images to the centers of Flot1-pits. Right: Radial intensity profiles of the averaged images shown on the left. Scale bar: 100 nm. (**H-**

J) Same as in (**E–G**), but for βII-spectrin (magenta) and exogenously expressed EndoA2 (green). (**K**) Percentages of Class I and Class II pits for exogenously expressed Flot1 and exogenously expressed EndoA2 in axons. Data are presented as mean ± s.e.m. ($n = 3$ biological replicates per condition). Boxplots show the median and boundaries (first and third quartile); Whiskers denote 1.5 times the interquartile range of the box. p-values calculated with two-sided paired Student's t-test.

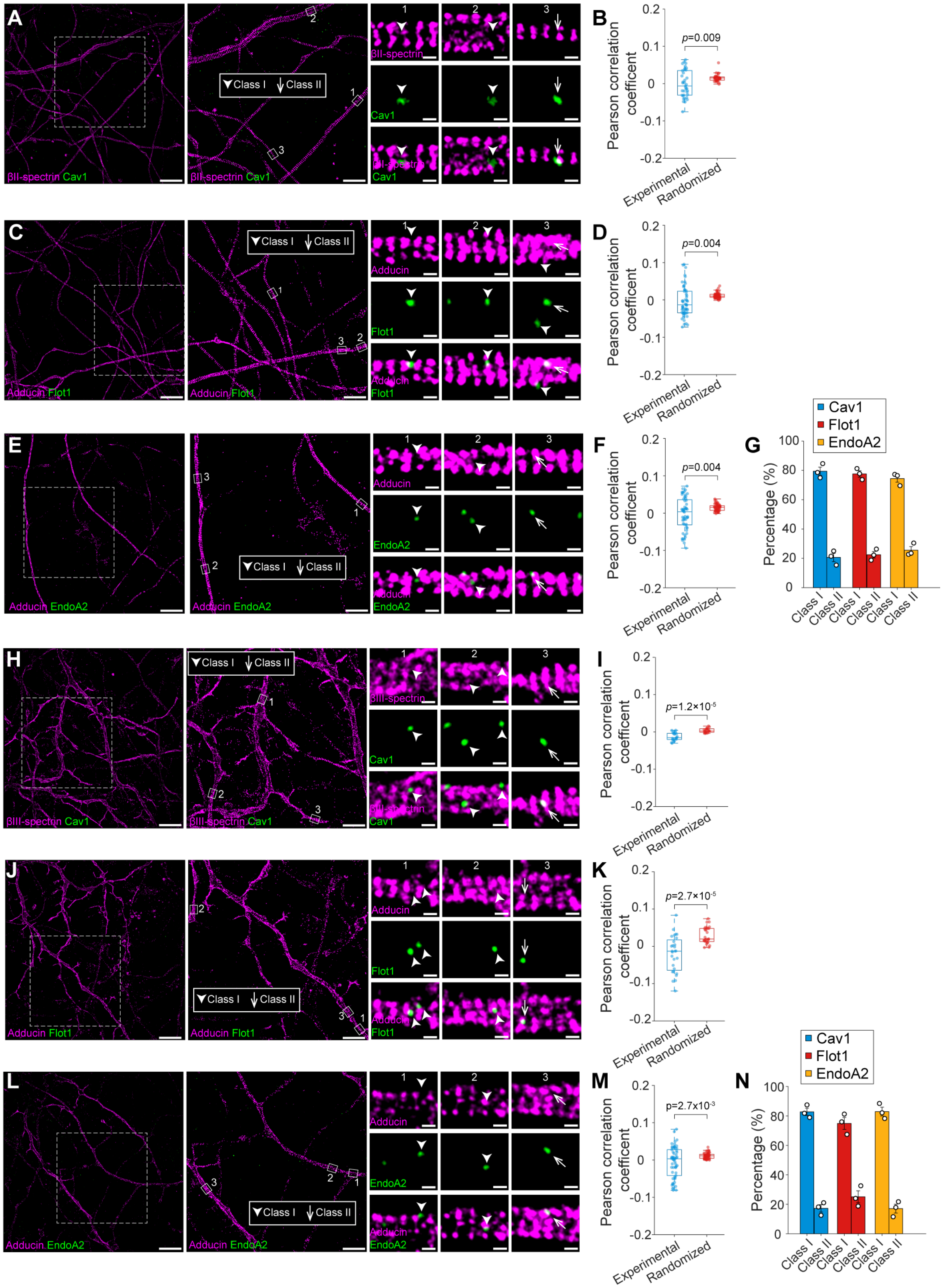

Fig. S5. Endogenous endocytic pits are localized within "clearings" of the MPS lattice in axons and dendrites, related to Fig. 2 and Fig. 3. (**A**) Left: Dual-color STORM images of βII-spectrin (magenta) and endogenous Cav1 (green) in axons. Right: Magnified views of Class I and Class II Cav1-pits in the

boxed regions. Scale bars: 10 μm (left), 5 μm (middle), 200 nm (right). (**B**) Pearson correlation coefficients between βII-spectrin and endogenous Cav1 under experimental and randomized conditions. (**C, D**) Same as (**A, B**), but for adducin (magenta) and endogenous Flot 1 (green). **(E, F**) Same as (**A, B**), but for adducin (magenta) and endogenous EndoA2 (green). **(G)** Percentages of Class I and Class II pits for endogenous Cav1, endogenous Flot1 and endogenous EndoA2 in axons. (**H**) Left: Dual-color STORM images of βIII-spectrin (magenta) and endogenous Cav1 (green) in dendrites. Right: Representative enlarged images of Class I and Class II Cav1-pits from the boxed regions. Scale bars: 10 μm (left), 5 μm (middle), 200 nm (right). (**I**) Pearson correlation coefficients between βIII-spectrin and endogenous Cav1 under experimental and randomized conditions. (**J, K**) Same as in (**H, I**), but for adducin (magenta) and endogenous Flot1 (green) in dendrites. (**L, M**) Same as in (**H, I**), but for adducin (magenta) and endogenous EndoA2 (green) in dendrites. (**N)** Percentages of Class I and Class II pits for endogenous Cav1, endogenous Flot1 and endogenous EndoA2 in dendrites. Data are presented as mean ± s.e.m. (n = 3 biological replicates per condition). Boxplots show the median and boundaries (first and third quartile); Whiskers denote 1.5 times the interquartile range of the box. p-values calculated with two-sided paired Student's t-test.

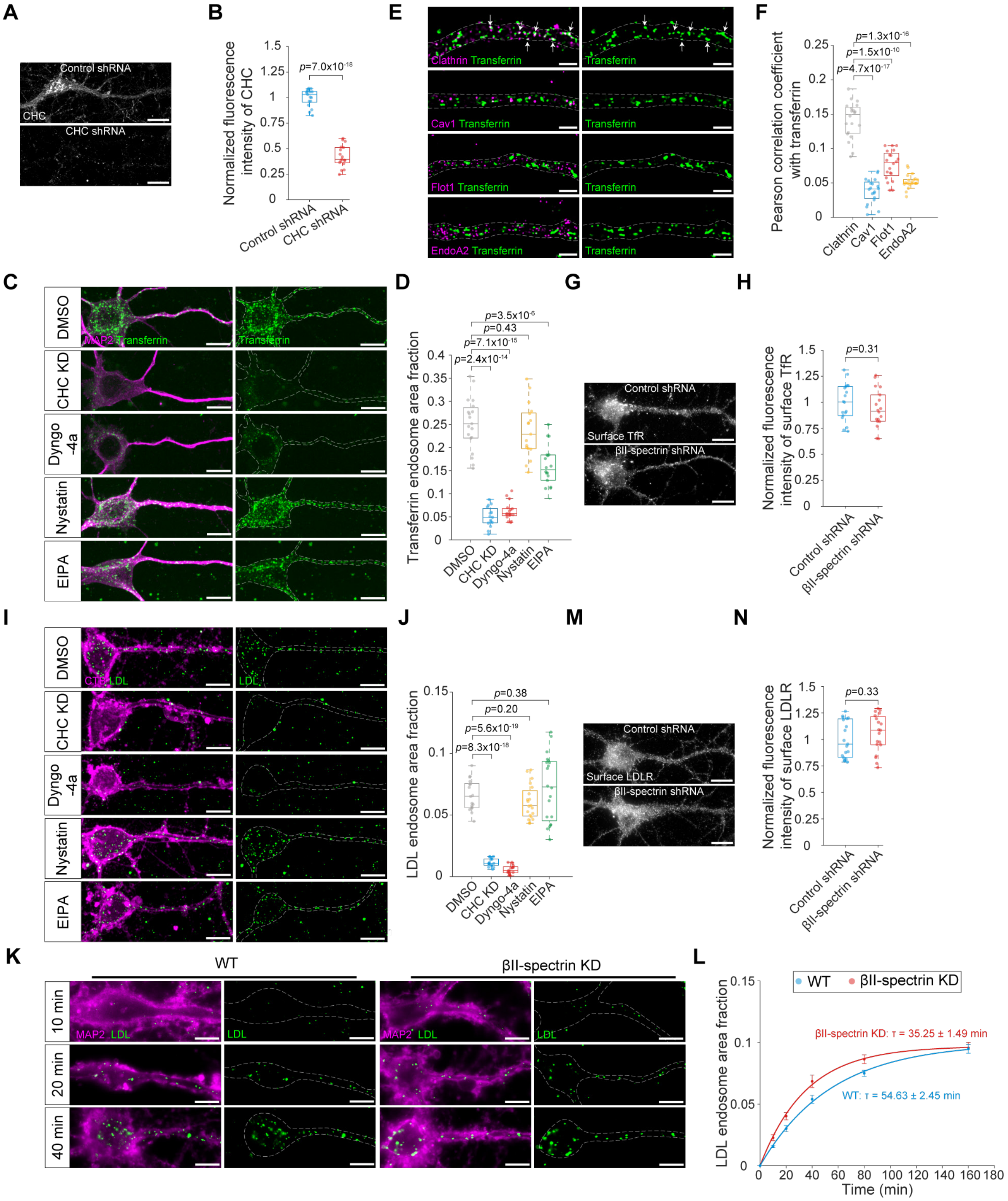

Fig. S6. Endocytic pathways for transferrin and LDL endocytosis and the effect of MPS disruption on LDL endocytosis, related to Fig. 4. (**A**) Widefield epi-fluorescence images of clathrin heavy chain (CHC) in neurons transduced with adenoviruses expressing either scrambled (control) shRNA or CHC shRNA. Scale bars: 25 μm. (**B**) Boxplots showing the normalized fluorescence intensity of CHC. (**C**) Confocal fluorescence images of MAP2 (magenta) and internalized CF568-transferrin (green) in somatodendritic region of CHC KD neurons pretreated with DMSO followed by CF568-transferrin treatment for 40 minutes, and WT neurons pretreated with DMSO, dyngo-4a, nystatin or EIPA for 30

minutes, followed by CF568-transferrin treatment for 40 minutes. Scale bars: 10 μm. (**D**) Boxplots of CF568-transferrin endocytosis in somatodendritic regions of neurons, quantified by the area fraction of transferrin endosomes. (**E**) Dual-color SIM images of internalized CF568-transferin (green) and endocytic pits (magenta; clathrin, Cav1, Flot1 or EndoA2) in dendrites of WT neurons treated with CF568-transferrin for 30 minutes. Regions of strong colocalization are indicated by white arrows. Scale bars: 2 μm. (**F**) Pearson correlation coefficients between internalized CF568-transferrin and endocytic pits. (**G**) Widefield epi-fluorescence images of surface-localized transferrin receptor (TfR) in WT and βII-spectrin KD neurons without ligand stimulation. Surface-localized TfR was immunostained with anti-TfR antibody following cell fixation without Triton permeabilization. Scale bars: 25 μm. (**H)** Boxplots showing the normalized fluorescence intensity of surface TfR. (**I**) Widefield epi-fluorescence images of CTB (magenta) and internalized Dil-LDL (green) in somatodendritic region of CHC KD neurons pretreated with DMSO, followed by treatment with Dil-LDL for 40 minutes, and WT neurons pretreated with DMSO, dyngo-4a, nystatin or EIPA for 30 minutes, followed by treatment with Dil-LDL for 40 minutes. Scale bars: 10 μm. (**J**) Boxplots of Dil-LDL endocytosis in somatodendritic regions of neurons, quantified by the area fraction of LDL endosomes. (**K**) Left: Widefield epi-fluorescence images of internalized Dil-LDL in somatodendritic regions of WT neurons treated with Dil-LDL for 10, 20 and 40 minutes. Right: Same as Left, but in βII-spectrin KD neurons. Scale bars: 10 μm. (**L**) Time course of LDL endocytosis in the somatodendritic regions of WT and βII-spectrin KD neurons, quantified by the area fraction of LDL endosomes. Solid lines represent single-exponential fits. Data are presented as mean ± s.e.m. (**M**) Widefield epi-fluorescence images of surface-localized LDLR in WT and βII-spectrin KD neurons without ligand stimulation. Surface-localized LDLR was immunostained with anti-LDLR antibody following cell fixation without Triton permeabilization. Scale bars: 25 μm. (**N**) Boxplots showing the normalized fluorescence intensity of surface LDLR. Boxplots show the median and boundaries (first and third quartile); Whiskers denote 1.5 times the interquartile range of the box. *p*-values calculated with two-sided unpaired Student's *t*-test.

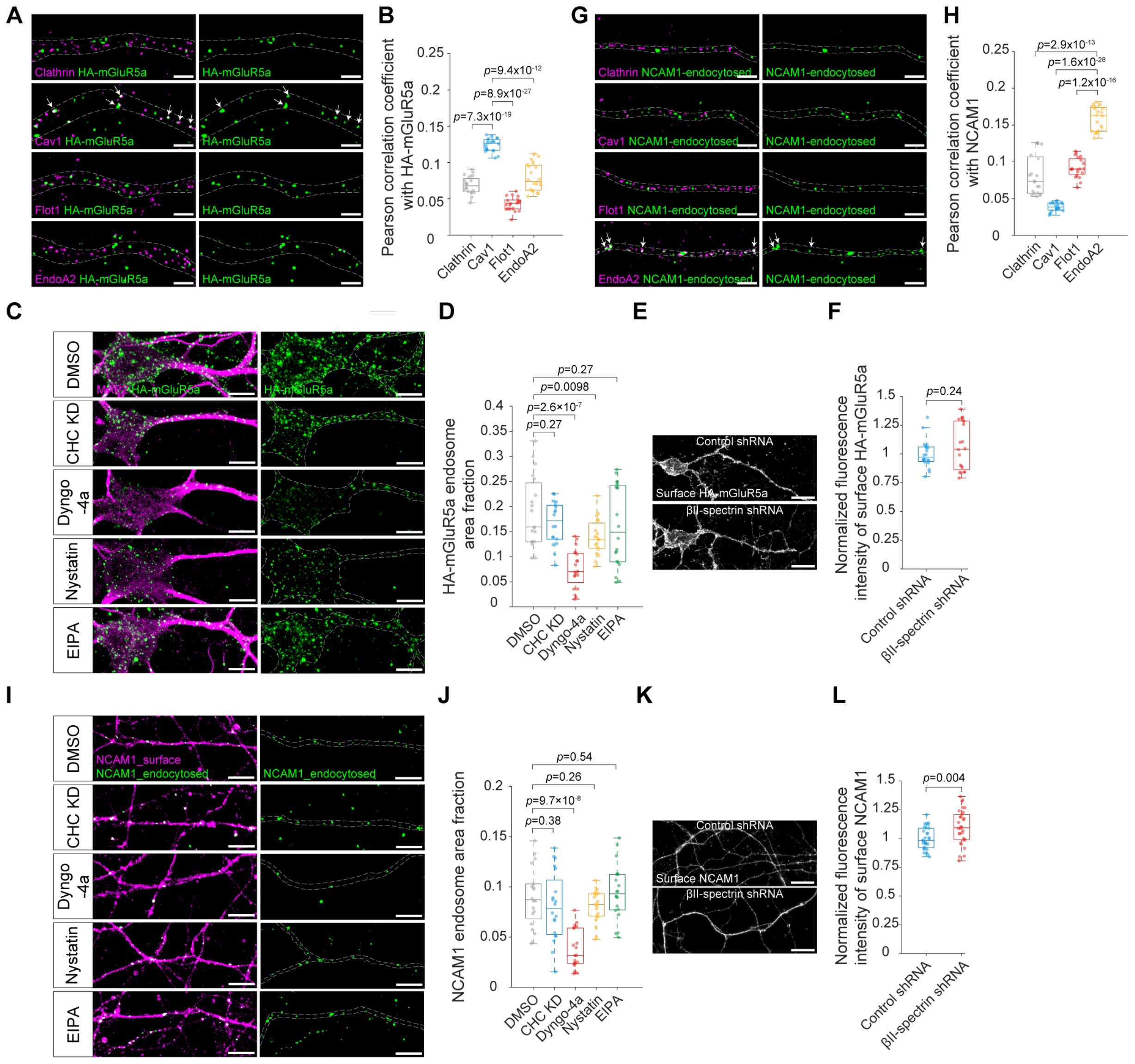

Fig. S7. Endocytic pathways for HA-mGluR5a and NCAM1 endocytosis, related to Fig. 4. (**A**) Dual-color SIM images of internalized HA-mGluR5a (green) and endocytic pits (magenta; clathrin, Cav1, Flot1 or EndoA2) in dendrites of WT neurons overexpressing HA-mGluR5a treated with anti-HA antibody for 30 minutes. Regions of strong colocalization are indicated by white arrows. Scale bars: 2 μm. (**B**) Pearson correlation coefficients between internalized HA-mGluR5a and endocytic pits. (**C**) Confocal fluorescence images of MAP2 (magenta) and internalized HA-mGluR5a (green) in the somatodendritic regions of CHC KD neurons overexpressing HA-mGluR5a pretreated with DMSO, followed by treatment with anti-HA antibody for 40 minutes, and WT neurons overexpressing HA-mGluR5a pretreated with DMSO, dyngo-4a, nystatin or EIPA for 30 minutes, followed by treatment with anti-HA antibody for 40 minutes. Scale bars: 10 μm. (**D**) Boxplots of HA-mGluR5a endocytosis in somatodendritic regions of neurons, quantified by the area fraction of HA-mGluR5a endosomes. (**E**) Widefield epi-fluorescence images of surface-localized HA-mGluR5a in WT and βII-spectrin KD neurons without ligand stimulation. Surface-localized HA-mGluR5a was immunostained with anti-HA antibody following cell fixation without Triton permeabilization. Scale bars: 25 μm. (**F**) Boxplots showing the normalized fluorescence intensity of surface HA-mGluR5a. (**G**) Dual-color SIM images of internalized NCAM1 (green) and endocytic pits (magenta; clathrin, Cav1, Flot1

or EndoA2) in axons of WT neurons treated with anti-NCAM1 antibody for 30 minutes. Regions of strong colocalization are indicated by white arrows. Scale bars, 2 µm. (**H**) Pearson correlation coefficients between internalized NCAM1 and endocytic pits. (**I**) Confocal fluorescence images of surface NCAM1 and internalized NCAM1 in CHC KD neurons pretreated with DMSO, followed by treatment with anti-NCAM1 antibody for 40 minutes, and WT neurons pretreated with DMSO, dyngo-4a, nystatin or EIPA for 30 minutes, followed by treatment with anti-NCAM1 antibody for 40 minutes. Scale bars: 10 µm. (**J**) Boxplots of NCAM1 endocytosis in neurons, quantified by the area fraction of NCAM1 endosomes. (**K**) Widefield epi-fluorescence images of surface-localized NCAM1 in WT and βII-spectrin KD neurons without ligand stimulation. Surface-localized NCAM1 was immunostained with anti-NCAM1 antibody following cell fixation without Triton permeabilization. Scale bars: 15 µm. (**L**) Boxplots showing the normalized fluorescence intensity of surface NCAM1. Boxplots show the median and boundaries (first and third quartile); Whiskers denote 1.5 times the interquartile range of the box. *p*-values calculated with two-sided unpaired Student's *t*-test.

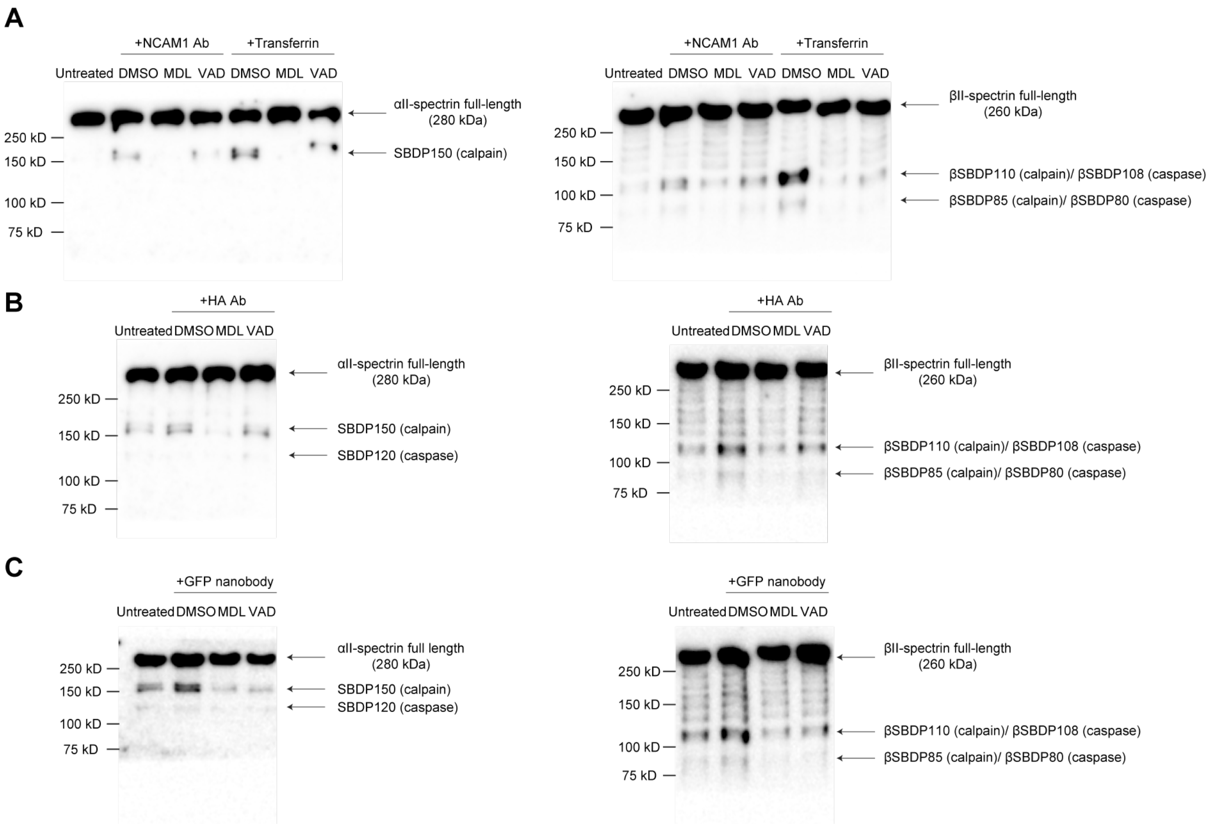

Fig. S8. Western blot analysis of MPS degradation triggered by ligand-induced endocytosis, related to Fig. 5. (**A**) Left: Immunoblot detection of αII-spectrin cleavage in WT neurons treated with anti-NCAM1 antibody or CF568-transferrin for 60 minutes. For inhibitor conditions, neurons were pretreated with MDL or VAD prior to ligand-induced endocytosis. Right: Same as Left, but for βII-spectrin cleavage. (**B**) Left: Immunoblot detection of αII-spectrin cleavage in neurons overexpressing HA-mGluR5a treated with anti-HA antibody for 60 minutes. For inhibitor conditions, neurons were pretreated with MDL or VAD prior to ligand-induced endocytosis. Right: Same as Left, but for βII-spectrin cleavage. (**C**) Left: immunoblot detection of αII-spectrin cleavage in neurons overexpressing SEP-APP treated with GFP nanobody for 60 minutes. For inhibitor conditions, neurons were pretreated with MDL or VAD prior to ligand-induced endocytosis. Right: Same as Left, but for βII-spectrin cleavage.

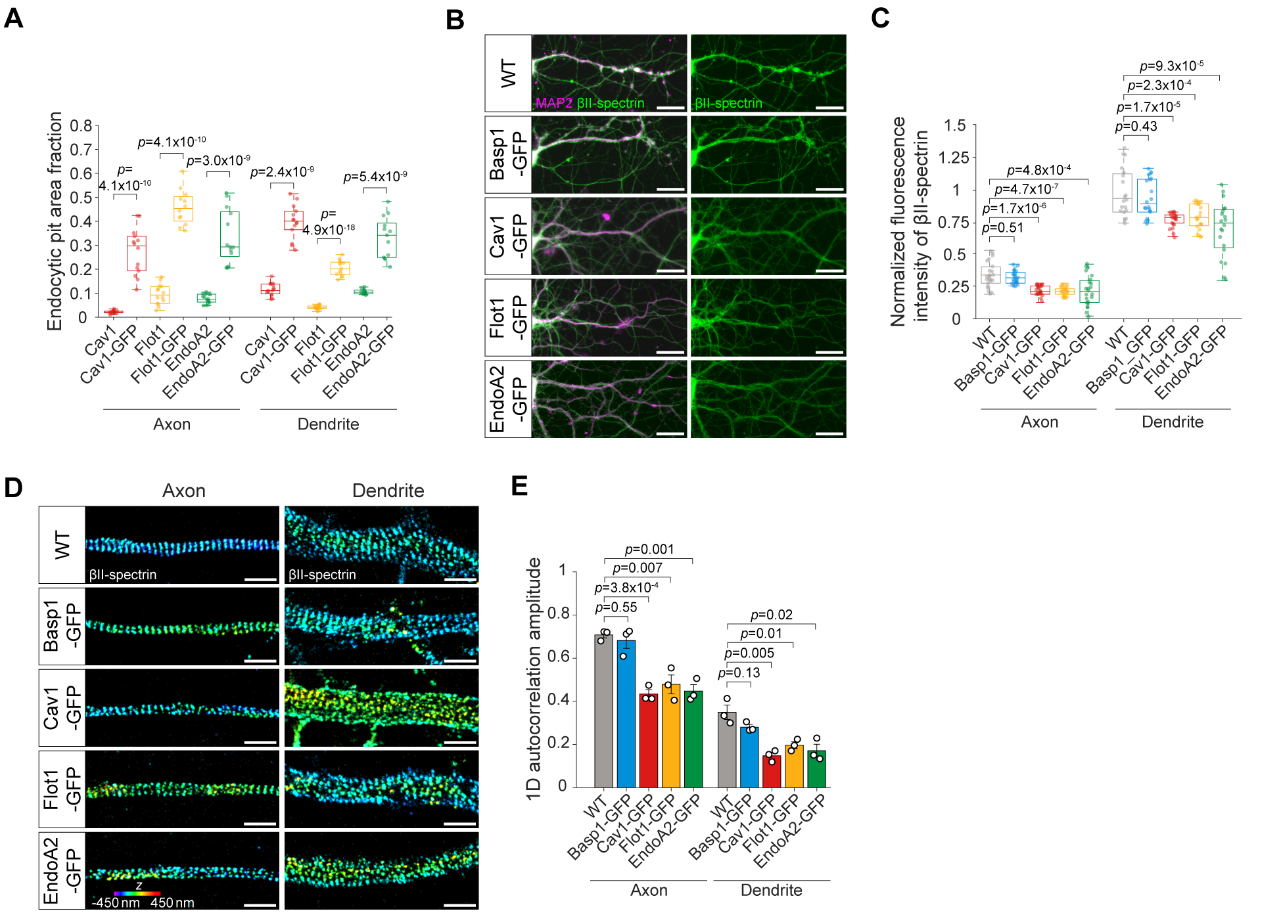

Fig. S9. Overexpression of endocytic proteins partially degrades the MPS in neurons, related to Fig. 5. (**A**) Boxplots of the area fraction of endogenous and overexpressed endocytic pits in axonal and dendritic neuronal compartments, quantified from the STORM data shown in Figs. 2 and 3, and Figs. S5. (**B**) Widefield epi-fluorescence images of MAP2 (magenta) and βII-spectrin (green) in WT neurons, and neurons overexpressing Basp1, Cav1, Flot1, and EndoA2. Scale bars, 25 µm. (**C**) Boxplots of normalized fluorescence intensity of βII-spectrin in axons and dendrites of neurons. (**D**) 3D STORM images of immunostained βII-spectrin in axons and dendrites of WT neurons, and neurons overexpressing Basp1, Cav1, Flot1, and EndoA2. Scale bars, 1 µm. Color scale bar represents the z-coordinate information. (**E**) Averaged 1D autocorrelation amplitude of βII-spectrin in axons and dendrites of neurons. Boxplots show the median and boundaries (first and third quartile); Whiskers denote 1.5 times the interquartile range of the box. *p*-values calculated with two-sided unpaired Student's *t*-test.

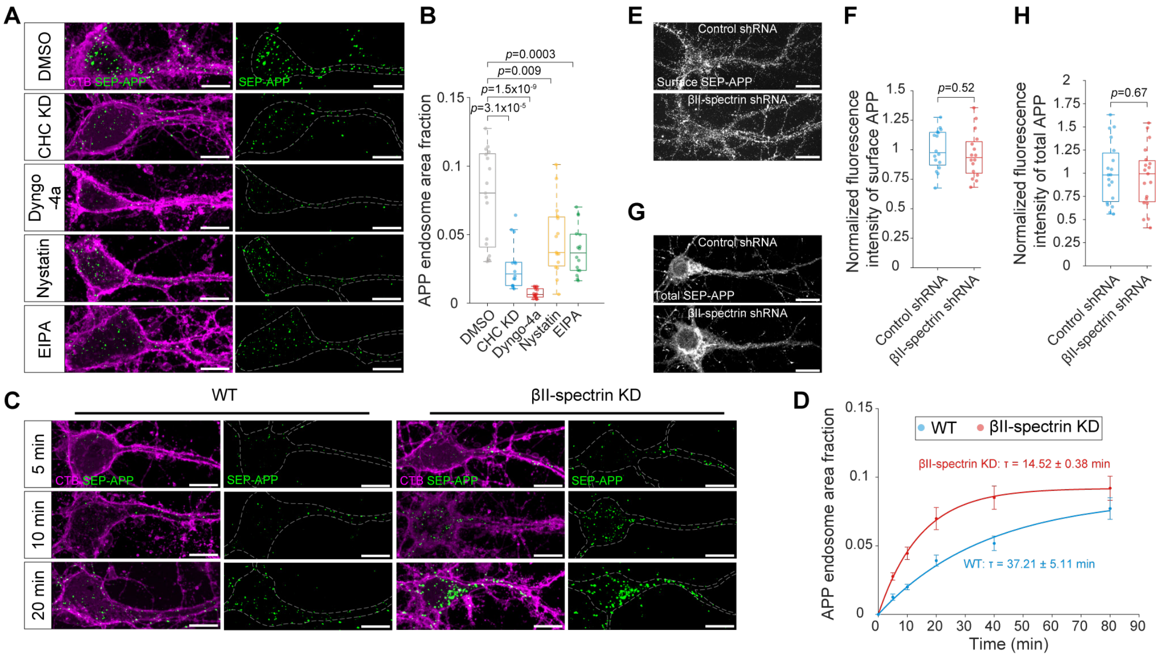

Fig. S10. Endocytic pathways of APP endocytosis, related to Fig. 6. (**A**) Confocal fluorescence images of CTB (magenta) and internalized SEP-APP (green) in somatodendritic regions of CHC KD neurons overexpressing SEP-APP pretreated with DMSO, followed by GFP nanobody treatment for 40 minutes, and WT neurons overexpressing SEP-APP pretreated with DMSO, dyngo-4a, nystatin, or EIPA for 30 minutes, followed by GFP nanobody treatment for 40 minutes. Scale bars: 10 µm. (**B**) Boxplots of SEP-APP endocytosis in somatodendritic regions of neurons, quantified by the area fraction of SEP-APP endosomes. (**C**) Left: Confocal fluorescence images of CTB (magenta) and internalized SEP-APP in somatodendritic region of WT neurons overexpressing SEP-APP treated with GFP nanobody for 5, 10, and 20 minutes. Right: Same as Left, but in βII-spectrin KD neurons. Scale bars: 10 µm. (**D**) Time course of APP endocytosis in the somatodendritic regions of WT and βII-spectrin KD neurons, quantified by the area fraction of APP endosomes. Solid lines represent single-exponential fits to the data. Data are presented as mean ± s.e.m. (**E**) Widefield epi-fluorescence images of surface-localized SEP-APP in WT and βII-spectrin KD neurons without ligand stimulation. Surface-localized SEP-APP was immunostained with anti-GFP antibody following cell fixation without Triton permeabilization. Scale bars: 25 µm. (**F**) Boxplots showing the normalized fluorescence intensity of surface SEP-APP. Boxplots show the median and boundaries (first and third quartile); Whiskers denote 1.5 times the interquartile range of the box. *p*-values calculated with two-sided unpaired Student's *t*-test. (**G**) Widefield epi-fluorescence images of total SEP-APP in WT and βII-spectrin KD neurons without ligand stimulation. Total SEP-APP was immunostained with anti-GFP antibody following cell fixation and Triton permeabilization. Scale bars: 25 µm. (**H**) Boxplots showing the normalized fluorescence intensity of total SEP-APP. Boxplots show the median and boundaries (first and third quartile); Whiskers denote 1.5 times the interquartile range of the box. *p*-values calculated with two-sided unpaired Student's *t*-test.
